# Neuronal *VPS35* deletion induces spinal cord motor neuron degeneration and early post-natal lethality

**DOI:** 10.1093/braincomms/fcab208

**Published:** 2021-09-10

**Authors:** Dorian Sargent, Lindsey A Cunningham, Dylan J Dues, Yue Ma, Jennifer J Kordich, Gabriela Mercado, Patrik Brundin, Rita M Cowell, Darren J Moore

**Affiliations:** 1 Department of Neurodegenerative Science, Van Andel Institute, Grand Rapids, MI 49503, USA; 2 Neuroscience, Drug Discovery Division, Southern Research, Birmingham, AL 35205, USA

**Keywords:** VPS35, retromer, motor neurons, neurodegeneration

## Abstract

Neurodegenerative diseases are characterized by the selective degeneration of neuronal populations in different brain regions and frequently the formation of distinct protein aggregates that often overlap between diseases. While the causes of many sporadic neurodegenerative diseases are unclear, genes associated with familial or sporadic forms of disease and the underlying cellular pathways involved tend to support common disease mechanisms. Underscoring this concept, mutations in the *Vacuolar Protein Sorting 35 Orthologue* (*VPS35*) gene have been identified to cause late-onset, autosomal dominant familial Parkinson’s disease, whereas reduced VPS35 protein levels are reported in vulnerable brain regions of subjects with Alzheimer’s disease, neurodegenerative tauopathies such as progressive supranuclear palsy and Pick’s disease, and amyotrophic lateral sclerosis. Therefore, VPS35 is commonly implicated in many neurodegenerative diseases. VPS35 plays a critical role in the retromer complex that mediates the retrieval and recycling of transmembrane protein cargo from endosomes to the *trans*-Golgi network or plasma membrane. VPS35 and retromer function are highly conserved in eukaryotic cells, with the homozygous deletion of *VPS35* inducing early embryonic lethality in mice that has hindered an understanding of its role in the brain. Here, we develop conditional knockout mice with the selective deletion of *VPS35* in neurons to better elucidate its role in neuronal viability and its connection to neurodegenerative diseases. Surprisingly, the pan-neuronal deletion of *VPS35* induces a progressive and rapid disease with motor deficits and early post-natal lethality. Underlying this neurological phenotype is the relatively selective and robust degeneration of motor neurons in the spinal cord. Neuronal loss is accompanied and preceded by the formation of p62-positive protein inclusions and robust reactive astrogliosis. Our study reveals a critical yet unappreciated role for VPS35 function in the normal maintenance and survival of motor neurons during post-natal development that has important implications for neurodegenerative diseases, particularly amyotrophic lateral sclerosis.

## Teaser

Sargent and Cunningham et al. report conditional knockout mice with pan-neuronal deletion of VPS35. The VPS35 conditional knockout mice exhibit motor deficits and early post-natal lethality that resulted from the selective degeneration of spinal cord motor neurons accompanied by marked reactive gliosis. Their findings may have important implications for amyotrophic lateral sclerosis.

## Graphical Abstract

**Graphical Abstract:**
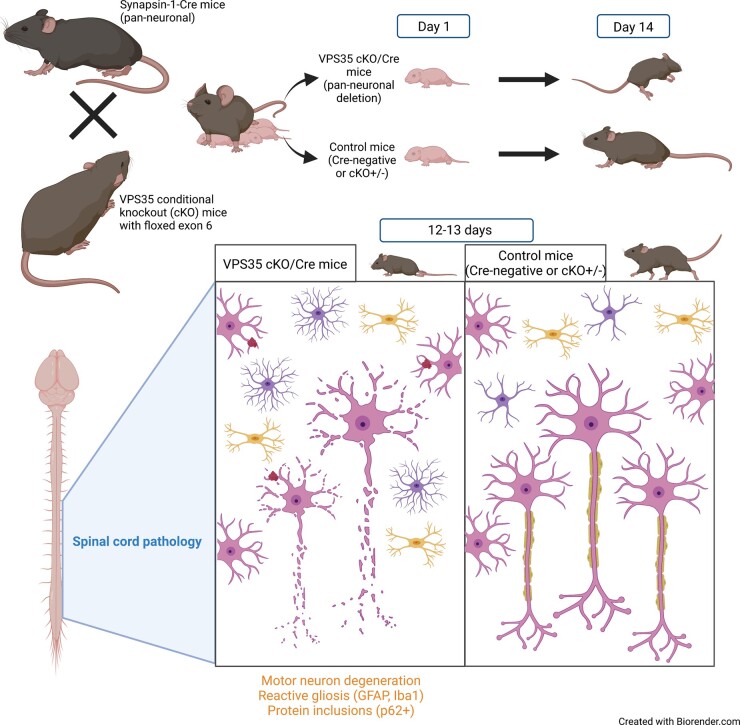

## Introduction

Neurodegenerative diseases, such as Parkinson’s disease, Alzheimer’s disease, frontotemporal dementias, tauopathies and amyotrophic lateral sclerosis (ALS), are characterized by the selective degeneration of distinct neuronal populations in different brain regions. The cause of neurodegeneration in common sporadic forms of these diseases remains uncertain, yet inherited forms of the disease have provided tremendous insight into underlying pathophysiological mechanisms. Familial forms of neurodegenerative diseases account for a small proportion of cases with rare causal mutations identified in a number of genes.^1–4^ Understanding the normal biological function of disease-linked gene products and the effects of familial mutations is important for identifying the molecular and cellular mechanisms underlying neurodegenerative disease. Gene products that span these distinct diseases are of particular interest for elucidating common pathophysiological mechanisms.

Vacuolar protein sorting 35 orthologue (VPS35) is a key subunit of the retromer complex, a heteropentameric complex implicated in the vesicular sorting and recycling of numerous transmembrane cargo proteins from endosomes to the *trans*-Golgi network or plasma membrane.^5–7^ The retromer is composed of two sub-complexes: the cargo-selective complex (containing the subunits VPS35, VPS29 and VPS26A or VPS26B) and a sorting nexin dimer. VPS35 binds to VPS26 at its N-terminus and VPS29 at its C-terminus and plays an important role in cargo recognition and binding, and accordingly is critical for retromer assembly and stability.^8^ VPS35 is therefore essential for normal retromer function and for organismal survival, since germline homozygous *VPS35* knockout mice exhibit early embryonic lethality.^9^ Emerging data indicate that VPS35 and the retromer play important roles in distinct neurodegenerative diseases.^10^ A heterozygous Asp620Asn (D620N) missense mutation in the *VPS35* gene causes a late-onset, autosomal dominant form of Parkinson’s disease that is clinically similar to sporadic Parkinson’s disease.^11^^,^^12^ Parkinson’s disease-like neuropathological features, including the degeneration of substantia nigra dopaminergic neurons and the accumulation of α-synuclein, have been observed in aged heterozygous *VPS35* knockout mice or in young mice with homozygous *VPS35* deletion selectively in dopaminergic neurons.^13^^,^^14^ These data suggest that Parkinson’s disease-linked mutations in *VPS35* could potentially manifest disease via a loss-of-function mechanism. Mechanistically, *VPS35* deficiency has been linked to mitochondrial impairment due to increased MUL1 and mitofusin-2 degradation,^14^ and defects in chaperone-mediated autophagy due to abnormal cargo sorting of the chaperone-mediated autophagy receptor LAMP2a,^13^ which is implicated in mediating the lysosomal degradation of mis-folded α-synuclein and other proteins.

We have recently described *D620N VPS35* knock-in mice that develop robust age-related nigral dopaminergic neurodegeneration, a key hallmark of Parkinson’s disease, as well as widespread axonal damage and tau-positive neuropathology.^15^ In contrast to *VPS35* null mice that are embryonic lethal, germline homozygous *D620N VPS35* knock-in mice exhibit normal viability and lifespan,^15^^,^^16^ implying that the D620N mutation does not manifest as a full loss of function. The connection of VPS35 with tau pathology in Parkinson’s disease models is intriguing, and VPS35 levels were shown to be reduced in vulnerable forebrain regions of subjects with different tauopathies, including Alzheimer’s disease, Pick’s disease and progressive supranuclear palsy.^17^^,^^18^ In agreement with these observations, recent studies in the rodent forebrain reveal that *VPS35* gene silencing is sufficient to induce tau pathology whereas pharmacological retromer stabilization is beneficial in an Alzheimer’s disease mouse model.^19^^,^^20^ Furthermore, in a mouse model of Alzheimer’s disease, *VPS35* heterozygosity worsens Alzheimer’s disease-like Aβ pathology by modulating BACE1 activity.^9^ A very recent study further implicates the retromer in ALS, with reduced levels of VPS35 and other subunits observed in spinal cord motor neurons from ALS subjects and G93A SOD1 mice, and novel pharmacological retromer stabilizers attenuating neurodegenerative phenotypes in these G93A mice.^21^ Collectively, these data provide support for a fundamental role for VPS35 and the retromer in regulating protein aggregation pathways and neuronal vulnerability broadly relevant to neurodegenerative diseases, including Parkinson’s disease, ALS, Alzheimer’s disease and related dementias characterized by tauopathy.

The physiological role of VPS35 and the retromer within different cells in the brain is poorly understood, as the bulk of prior mechanistic studies are based upon observations in yeast and mammalian cell lines. Furthermore, exploring the impact of *VPS35* deficiency in the brain has been complicated by embryonic lethality in germline knockout mice.^9^^,^^15^ Recently, *VPS35* depletion specifically in microglia was shown to impair hippocampal neurogenesis and cause depression-like behaviour in mice but is not sufficient to cause neurodegeneration or alter survival.^22^ While the detrimental effects of *VPS35* depletion have so far been reported in specific neuronal populations, including dopaminergic neurons or embryonic cortical pyramidal neurons,^14^^,^^23^ the overall impact of *VPS35* depletion in neurons in general is not known. This is important for understanding the selective vulnerability of different neuronal populations to *VPS35* deficiency that will provide key insight into neuronal susceptibility in different neurodegenerative diseases. Accordingly, here we generate conditional knockout (cKO) mice with the selective pan-neuronal deletion of *VPS35* by crossing cKO mice to synapsin-1-Cre transgenic mice. Surprisingly, the neuronal-specific deletion of *VPS35* produces a progressive disease with motor deficits and early post-natal lethality that is pathologically characterized by the selective degeneration of ventral horn motor neurons in the spinal cord accompanied by protein inclusions and marked gliosis. Our data reveal that VPS35 is critical for the normal maintenance and survival of motor neurons during post-natal development that has implications for neurodegenerative diseases, particularly ALS and related disorders.

## Materials and methods

### Analysis of mouse brain single-cell transcriptome datasets

Two single-cell transcriptome databases were used to explore VPS35 mRNA expression in the mouse central nervous system: Mousebrain.org^24^ and Dropviz.org.^25^ For Mousebrain.org data, expression values and metadata per cluster files were downloaded from the website and accessed using loomR version 0.2.0 (https://satijalab.org/loomR/loomR_tutorial.html, last accessed May 6 2020). Data were categorized into cell-type-specific categories using the clusters identified by Ref.^24^ For Dropviz.org data, subcluster metacell data representing aggregate Unique Molecular Identifier counts were downloaded, and subcluster data for VPS35 counts were divided by the total Unique Molecular Identifiers per subcluster to generate relative abundance values. The cell-type-specific identity of subclusters was defined using the previous characterization of subclusters as a guide.^25^ For both databases, values were normalized to the highest expressing cluster to enable comparison across all cell types within that database. Clusters or subclusters with one or two datapoints per cell type were not included in the analyses. Datasets were visualized and analysed in GraphPad Prism v.8 using violin plots and a non-parametric one-way ANOVA test (Kruskal–Wallis test) followed by a Dunn’s multiple comparison test, with significance set at *P* < 0.05.

### Animals

All animal experiments were approved by the Van Andel Institute Institutional Animal Care and Use Committee and conducted in strict accordance with the National Institutes of Health (NIH) Guide for the Care and Use of Laboratory Animals (National Research Council Committee for the Update of the Guide for the and Use of Laboratory 2011). Animals were provided with food and water *ad libitum* and exposed to 12 h light/dark cycle and maintained in a pathogen-free barrier facility. PGK1-FLPo (stock no. 011065), Synapsin-1-Cre (Syn1-Cre; stock no. 003966) and mT/mG reporter [*ROSA26^tm^4^(ACTB-tdTomato,-EGFP)^*; stock no. 007676] mice were purchased from the Jackson Laboratory.

### Generation of conditional *VPS35* knockout mice

*VPS35^flox/flox^* (cKO) mice were generated from gene-targeted embryonic stem (ES) cells purchased from the International Mouse Phenotyping Consortium [clone D11, strain *Vps35^tm1a(EUCOMM)Hmgu^*]. *VPS35^tm1a(EUCOMM)Hmgu^* ES cells (JM8A3.N1 parental line) derived from C57BL/6N-A/a mice containing a KO-first allele (reporter-tagged insertion with conditional potential) were injected into the inner cell mass of C57BL/6N blastocysts for implantation into pseudo-pregnant foster mothers. The resulting chimeric progeny were bred to C57BL/6N mice to identify founders producing germline transmission by PCR genotyping of genomic tail DNA. Founder mice harbour a KO-first allele containing an LacZ-neo cassette flanked by *FRT* sites and a *loxP*-flanked exon 6 within the *VPS35* gene. The KO-first allele disrupts the normal expression of VPS35, effectively creating a *VPS35* heterozygous null animal (*VPS35^+/−^*) that is unable to become homozygous due to embryonic lethality. To remove the *FRT*-flanked LacZ-neo cassette, KO-first mice were crossed to PGK1-FLPo mice (stock no. 011065, The Jackson Laboratory). Removal of the LacZ-neo cassette in progeny was confirmed by genomic PCR, the restoration of normal VPS35 protein levels and by the ability to generate viable homozygous *VPS35^flox/flox^* mice, as previously described.^14^  *VPS35^+/^*^*−*^*/FLPo^+^* mice were crossed to C57BL/6J mice in order to remove the *FLPo* gene, finally producing *VPS35^flox/+^* mice harbouring a conditional floxed exon 6. Intercrossing of *VPS35^flox/+^* progeny produced homozygous *VPS35^flox/flox^* mice that exhibit normal viability and survival.

**Figure 1:**
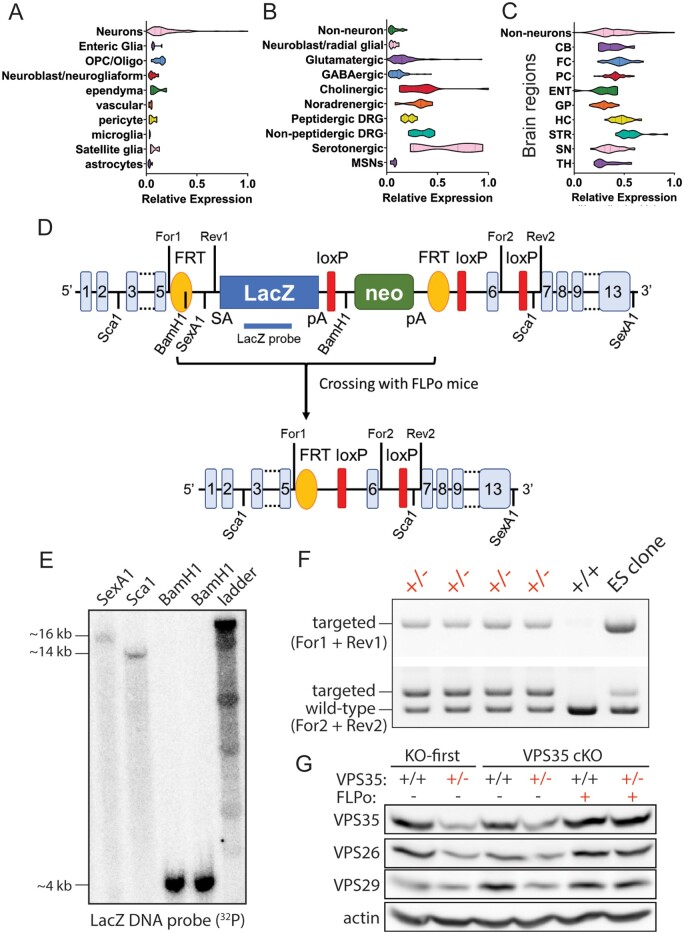
**Generation of *VPS35* conditional knockout mice.** (**A–C**) Single-cell RNAseq datasets derived from mousebrain.org (**A, B**) and dropviz.org (**C**), indicating VPS35 expression in different cell types of mouse brain (**A**), different neuronal populations (**B**) or neuronal expression of *VPS35* in different brain regions (**C**). DRG = dorsal root ganglion; MSN = medium spiny neurons; CB = cerebellum; FC = frontal cortex; PC = parietal cortex; ENT = entorhinal cortex; GP = globus pallidus; HC = hippocampus; STR = striatum; SN = substantia nigra; TH = thalamus. Statistical comparisons are shown in Supplementary Tables 1–3. (**D**) Schematic indicating the gene-targeted *VPS35* locus in mice derived from *Vps35^tm1a(EUCOMM)Hmgu^* ES cells (KO-first allele), and the expected impact of *FLPo*-mediated recombination to produce a conditional *VPS35* locus with *loxP*-flanked exon 6. The location of key restriction sites, PCR primers used for genotyping of 5ʹ (For1/Rev1) and 3ʹ (For2/Rev2) ends, as well as a LacZ probe used for Southern blot analysis, are indicated. (**E**) Southern blot analysis of restriction-digested genomic DNA derived from *Vps35^tm1a(EUCOMM)Hmgu^* ES cells using a [^32^P]-labelled LacZ-specific DNA probe. (**F**) PCR analysis of tail genomic DNA from heterozygous *VPS35* KO-first (+/−) or wild-type (+/+) mice using 5ʹ or 3ʹ primer pairs. (**G**) Western blot analysis of soluble hemi-brain extracts derived from *VPS35* KO-first mice and following crossing with FLPo mice to produce *VPS35* cKO mice, indicating levels of endogenous VPS35, VPS26, VPS29 and actin. Retromer subunits are reduced in *VPS35* KO-first mice (+/−) but restored to normal levels in cKO mice (+/−) containing FLPo.

**Figure 2:**
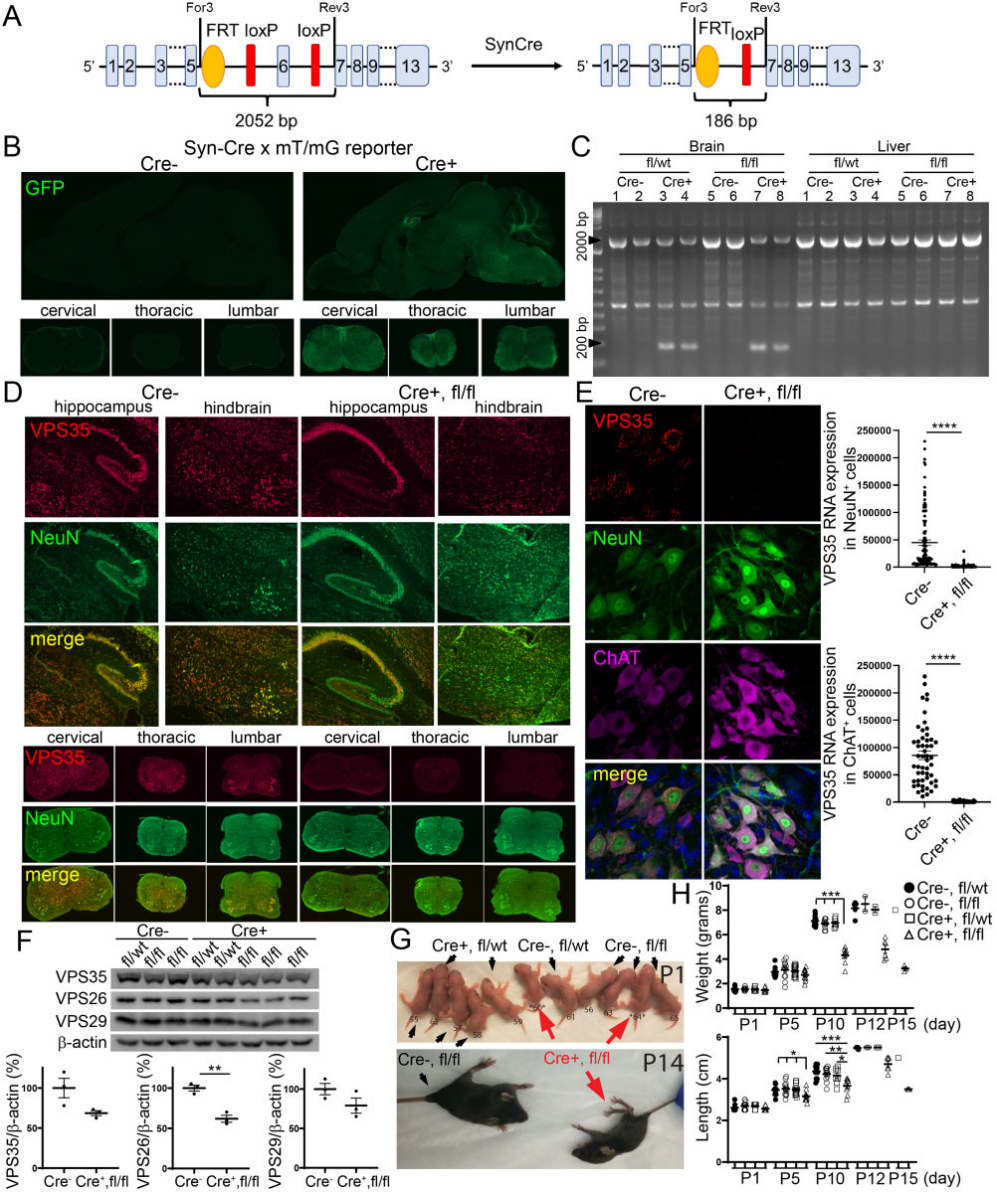
**Molecular and phenotypic analyses of *VPS35* cKO mice crossed with Synapsin-1-Cre mice.** (**A**) Schematic indicating the targeted *VPS35* cKO allele before or after Cre-mediated recombination (with synapsin-1-Cre mice) to remove exon 6 selectively in neurons. The location of PCR primers used for detecting genomic recombination in **C** is shown. (**B**) Fluorescent images indicating GFP (green) in sagittal sections of brain (*upper*) and coronal sections of the spinal cord (*lower*) induced by Cre-mediated recombination in mT/mG reporter mice crossed with Syn1-Cre mice at P10. (**C**) PCR analysis of genomic DNA derived from brain or liver of *VPS35* cKO mice (floxed, fl) crossed with Syn1-Cre at P10 using primers flanking exon 6 (For3/Rev3) shown in **A**. (**D**) Representative images of VPS35 mRNA (red) detected by fluorescence *in situ* hybridization (RNAscope) co-localized with NeuN immunofluorescence (green) in the hippocampus and hindbrain (*upper panels*) or the cervical, thoracic and lumbar spinal cord (*lower panels*) of *VPS35^fl/fl^/Cre* mice at P10 compared to Cre-negative mice. (**E**) Confocal microscopic images of VPS35 mRNA (red) detected by RNAscope co-localized with NeuN (green) and ChAT (purple) immunofluorescence indicating VPS35 mRNA expression in ventral horn motor neurons of the lumbar spinal cord in P10 mice. Quantitation of VPS35 mRNA fluorescence intensity in NeuN-positive neurons (*n* = 113 cells, from three Cre-negative mice; *n* = 125 cells, from three *VPS35^fl/fl^/Cre* mice) and ChAT-positive neurons (*n* = 52 cells, from three Cre-negative mice; *n* = 58 cells, from three *VPS35^fl/fl^/Cre* mice) localized within the dorsal and ventral horns of the lumbar spinal cord is shown (*right*). Bars represent the mean ± SEM. ****P < 0.0001 by unpaired, two-tailed Student’s *t*-test. (**F**) Levels of VPS35, VPS26, VPS29 and actin proteins in soluble spinal cord extracts of *VPS35* cKO mice at P12–P14 by western blot analysis. Graphs indicate densitometric quantification of VPS35, VPS26 or VPS29 levels normalized to actin from *VPS35^fl/fl^/Cre* mice (*n* = 3 mice) compared to Cre-negative control mice (*VPS35^fl/fl^* or *VPS35^fl/wt^*; *n* = 3 mice). Bars represent the mean ± SEM. **P < 0.01 by unpaired, two-tailed Student’s *t*-test. (**G**) Representative images of mouse littermates at P1 (*upper*) or P14 (*lower*) with their indicated genotypes. *VPS35^fl/fl^/Cre* mice are indicated by red arrows. (**H**) Graphs indicating body weight (grams, *upper*) and body length (cm, *lower*) of mice at P1, P5, P10, P12 or P15 for each genotype. Line bars represent mean ± SEM (*n* = 8–18 mice/genotype for P1, *n* = 11–15 mice for P5, *n* = 8–14 mice for P10). Note, *n* = 2–5 mice/genotype for P12 or P15 due to limited surviving animals (with error bars present for *n* ≥ 2). Data points are shown for individual mice as indicated by black or white shapes. White triangles indicate *VPS35^fl/fl^/Cre* mice. *P < 0.05, **P < 0.01, ***P < 0.001 by one-way ANOVA with Bonferroni’s *post hoc* test, compared to *VPS35^fl/wt^* mice (black circles) or as indicated.

To generate neuron-specific *VPS35* KO mice for these studies, *VPS35^flox/flox^* mice were initially crossed with Syn1-Cre mice to produce *VPS35^flox/+^*/Syn1-Cre progeny that were subsequently bred with *VPS35^flox/flox^* mice to produce four genotypes of mixed sex at an expected frequency of 25%: *VPS35^flox/+^*, *VPS35^flox/+^*/Syn1-Cre, *VPS35^flox/flox^* and *VPS35^flox/flox^*/Syn1-Cre. To monitor health status, mice from all four genotypes were evaluated for survival, weight and body length up to P16. Hemizygous Syn1-Cre^Tg/+^ mice were also crossed with heterozygous mT/mG reporter mice to monitor neuronal Cre expression/activity throughout the brain of neonatal and adult mice. For all mouse litters, genomic DNA derived from tail biopsies was used for PCR to identify mouse genotypes, as recommended for each mouse strain from JAX Labs. For distinguishing the genotypes of *VPS35* KO-first and floxed mice, we used a combination of PCR primer pairs flanking the 5ʹ end of the LacZ-neo cassette (For1: 5′-AAGCCATCATGCTCAGGAGT-3′, Rev1: 5′-CCCTTCAGTCTTCCTGTCCA-3′) producing a 467 bp product for the KO-first allele, and primers flanking the 3ʹ *loxP* site (For2: 5′-AACCAGCTCCCAACAAAATG-3′, Rev2: 5′-TGATCAAGGGAGAGGGAGAA-3′) producing 339 bp (wt mice) and 404 bp (floxed or KO-first mice) products, as indicated in Figs 1D and F. To confirm brain-restricted Cre-mediated recombination at the targeted *VPS35* locus in *VPS35^flox/flox^*/Syn1-Cre mice and their littermates (Figs 2A and C), we purified genomic DNA from brain and liver samples using the DNeasy Blood & Tissue Kit (Qiagen). Genomic DNA was subjected to PCR using primer pairs located in flanking regions of exon 6 (For3: 5′-CCGAAGTTCCTATTCCGAAG-3′, Rev3: 5′-AAATGTGAGTGGGACCAAGC-3′) producing a 186 bp product with recombination (Cre+, lacking exon 6) and/or a 2052 bp product without recombination (Cre-, containing exon 6), as shown in Fig. 2A. Exon 6 excision could only be detected in brain tissue from *VPS35^flox/flox^*/Syn1-Cre and *VPS35^flox/wt^*/Syn1-Cre mice, but not in liver.

### Tissue preparation

Mouse pups (≤P15) were euthanized by injection with a lethal overdose of tribromoethanol (Avertin) and then decapitated. Brain and spinal cord were dissected and immersion-fixed in 4% paraformaldehyde overnight at 4°C. Paraformaldehyde was replaced by 30% sucrose in PBS (phosphate-buffered saline) for cryopreservation of brain tissues. Fixed tissues were flash-frozen for sectioning on a microtome (SM2010R, Leica) into 35-μm-thick sagittal (brain) and coronal (spinal cord) sections. Sections were collected and stored in cryoprotectant solution (30% sucrose, 30% ethylene glycol, in 0.1 M phosphate buffer) until use.

### Antibodies

For western blot analysis, the following primary antibodies were used: mouse monoclonal anti-VPS35 (ab57632, Abcam), rabbit polyclonal anti-VPS26 (ab23892, Abcam), goat polyclonal anti-VPS29 (ab10160, Abcam), rabbit monoclonal anti-pThr73-Rab10 (clone MJF-R21, ab230261, Abcam), rabbit monoclonal anti-Rab10 (clone D36C4, #8127, Cell Signaling Tech.) or mouse monoclonal anti-actin (clone C4, MAB1501, Millipore).

For immunohistochemistry or immunofluorescence the following primary antibodies were used: rabbit monoclonal anti-VPS35 (ab157220, Abcam), rabbit polyclonal anti-NeuN (ABN78, Millipore), rabbit monoclonal anti-choline acetyltransferase (ab178850, Abcam), rabbit monoclonal anti-active caspase-3 (clone C92-605, 559565, BD Biosciences), guinea pig polyclonal anti-p62 (GP62-C, Progen), rabbit polyclonal anti-microtubule-associated protein 2 (M3696, Sigma), rabbit polyclonal anti-LAMP1 (ab24170, Abcam), mouse monoclonal anti-phospho-Ser202/Thr205-tau (clone AT8, MN1020, ThermoFisher), mouse monoclonal anti-tau conformation-specific (clone MC1, kindly provided by Peter Davies, Feinstein Institute for Medical Research, New York), rabbit polyclonal anti-TDP-43 (10782–2-AP, ProteinTech), mouse monoclonal anti-α-synuclein (clone Syn1, 610787, BD Biosciences), mouse monoclonal anti-C9ORF72 (CTX634482, GeneTex), rabbit polyclonal anti-glial fibrillary acidic protein (E18320, Spring Bioscience) or rabbit polyclonal anti-Iba1 (019–19741, Wako).

Secondary anti-mouse, anti-rabbit or anti-guinea pig, horse radish peroxidase (HRP)-conjugated IgG, light chain-specific (Jackson ImmunoResearch), biotinylated IgG (Vector Labs) or AlexaFluor488, −594 or −647 IgG (ThermoFisher) were used.

### RNAscope-fluorescence *in situ* hybridization

Fluorescence *in situ* RNA hybridization was performed using an RNAscope Multiplex Fluorescent Detection v2 kit (Cat. No. 323100; Advanced Cell Diagnostics) combined with an Opal™ 570 fluorophore (FP1488001KT Akoya Biosciences) according to the manufacturer’s instructions. Fixed brains were flash-frozen to prepare 12 μm-thick sagittal sections using a cryostat (Leica) and mounted on SuperFrost plus slides (Fisher Scientific). An RNAscope Target Probe recognizing nucleotides 35-1193 (NCBI Ref. Seq. NM_022997.4) of mouse VPS35 mRNA (Cat No. 431071; Advanced Cell Diagnostics) was used. Immunofluorescence was performed following *in situ* hybridization with anti-NeuN and anti-choline acetyltransferase (ChAT) primary antibodies, and AlexaFluor-488 and -647 anti-IgG secondary antibodies. Levels of fluorescence specific to VPS35 mRNA were measured from 60× confocal images using ImageJ. Corrected total cell fluorescence values were calculated in NeuN+/ChAT+, NeuN-/ChAT+, NeuN+/ChAT- neuronal populations sampled from ventral and dorsal horns of lumbar spinal cord sections.

### Immunofluorescence and confocal microscopy

Tissue sections were blocked in 1X PBS pH 7.4 containing 0.2% Triton-X100, 10% normal goat serum, 0.4% BSA. Sections were incubated overnight at 4°C with primary antibodies in 1X PBS containing 0.1% Triton-X100 and 0.4% BSA. After 3× washes with 0.1% Triton-X100 in PBS, sections were incubated with AlexaFluor-conjugated secondary antibodies in 0.3% Triton-X100, 0.4% BSA in PBS. Finally, sections were washed 3× times in 0.1% Triton-X100 in PBS before being mounted on slides using Fluoromount-G medium containing DAPI (ThermoFisher). Fluorescence images were acquired using an Olympus BX63 fluorescence microscope (Olympus) or a Nikon A1plus-RSi laser-scanning confocal microscope (Nikon Instruments) equipped with a 60× oil objective. Confocal images were subjected to deconvolution using Huygens Professional software (Scientific Volume Imaging). Fluorescent ChAT-positive motor neurons within both ventral horns were counted manually in the thoracic and lumbar spinal cord, using anatomically matched and serial sections (six sections per animal), by an experimenter blinded to mouse genotype. One group member not involved in this study was responsible for blinding of samples prior to counting and unblinding of samples after counting prior to data analysis. The number of p62-positive inclusions throughout spinal cord sections was quantified using similar manual counting methodology as above.

### Immunohistochemistry and histology

Antigen retrieval was performed on tissues for selected antibodies (VPS35, and active caspase-3). For active caspase-3 staining, sections were mounted on slides and incubated overnight in a 1:1 ethanol:chloroform solution to reduce background fat/lipid staining. Sections were rehydrated and antigen retrieval was performed in Universal HIER antigen reagent (ab208572, Abcam) at 95–100°C for 30 min.

For immunohistochemistry, sections were incubated in a 0.3% H_2_O_2_ solution at 4°C for 10 min to quench endogenous peroxidase activity. Sections were washed 2× in 0.1% Triton-X100 in 1X TBS pH 7.4 (TBST), and sections were blocked by incubation with 10% normal goat serum (Invitrogen) and 0.1% Triton-X100 in 1X PBS pH 7.4 for 1 h at room temperature. Sections were incubated overnight at 4°C with each primary antibody in 1X PBS containing 5% normal goat serum. After washing in TBST, sections were incubated with biotinylated secondary antibodies (Vector Labs) in TBST for 2 h at room temperature, followed by Avidin-Biotin Complex (ABC) reagent (Vector Labs) for 1 h at room temperature, and visualized by incubation with 3,3ʹ-diaminobenzidine tertrahydrochloride reagent (Vector Labs). Sections were mounted onto Superfrost plus slides (Fisher Scientific), dehydrated with increasing ethanol concentrations and xylene, and coverslipped using Entellan mounting medium (Merck). Brightfield images were captured using an Axio Imager M2 upright microscope (Zeiss) equipped with a colour CCD camera.

Gallyas silver staining was conducted with the FD NeuroSilver kit II (PK301A, FD Neurotechnologies) according to the manufacturer’s instructions. An additional 8 min was added to the incubation step in Solution D + E, which allowed the detection of dark degenerating cell bodies. Images for all genotypes were captured together using an Aperio ScanScope XT slide scanner. Glial fibrillary acidic protein (GFAP) and Iba1 immunostaining was also imaged by slide scanner and quantified using HALO analysis software (Indica Labs) with the same algorithm and threshold applied across slides and genotypes. NeuN-immunostained spinal cord sections to monitor total area were captured and analysed using Halo software. Nissl-positive dark, condensed motor neurons within both ventral horns were counted manually in the thoracic and lumbar spinal cord, using anatomically matched and serial sections (six sections per animal), by an experimenter blinded to mouse genotype during counting.

### Western blot analysis

Spinal cord samples were homogenized in lysis buffer [50 mM Tris-HCl, pH 7.5, 1 mM EDTA, 1 mM EGTA, 1% Triton-X100, 5% glycerol, Phosphatase Inhibitor Cocktail Set 1 (Sigma), 0.2 mM sodium orthovanadate (Sigma), 10 mM sodium fluoride (VWR chemical), 2 mM 2-glycerophosphate (Chem-Impex International), 2 mM sodium pyrophosphate (Sigma), Complete™ Mini Protease Inhibitor Cocktail (Roche)] using an T10 Ultra-Turrax mechanical disperser (IKA). Homogenates were clarified by centrifugation at 15 000 *g* for 30 min at 4°C and soluble extracts were quantified by BCA assay (ThermoFisher Scientific). Protein extracts were diluted in Laemmli sample buffer and resolved on 7.5% or 12.5% sodium dodecyl sulfate-polyacrylamide gel electrophoresis (SDS-PAGE) gels, followed by transfer to 0.2 µm nitrocellulose membranes (Protran, GE Healthcare). Membranes were blocked in 5% milk in 1× TBS containing 0.1% Tween-20 (TBST) for 1 h at room temperature before incubation overnight at 4°C with primary antibodies and for 1 h with HRP-conjugated light chain-specific anti-IgG (Jackson Immunoresearch) in TBST. Proteins were visualized by enhanced chemiluminescence (ECL reagent, GE Healthcare) and imaged on an Amersham Imager 680 (GE Healthcare). Quantification of protein bands by densitometry was conducted using Image Studio™ Lite v5.2 software (LI-COR Biosciences).

### Data availability

All relevant data are presented in this manuscript and no large numerical datasets, algorithms or software were generated as part of this study.

### Statistical analyses

The sample size was estimated based on our prior mouse studies to achieve *n* ≥ 5 mice per genotype. No criteria were applied for excluding animals during the experiments or data points during the analysis. Criteria for the inclusion of animals were based on genotype. Potential confounders were not controlled. One-way ANOVA with *post*  *hoc* was used for multiple comparisons, and unpaired, two-tailed Student’s *t*-test was used for comparisons between two groups. All data were plotted as mean ± SEM. *P* < 0.05 was considered significant. Statistical analysis was conducted using statistical functions included in GraphPad Prism software, where all data met the assumptions of the test selected.

## Results

### Generation and molecular characterization of *VPS35* conditional knockout mice

We first set out to explore the cellular and regional distribution of endogenous VPS35 within the mouse brain. Since immunohistochemical analyses in brain tissue with VPS35-specific antibodies have proven challenging, we analysed public repositories containing single-cell RNA-Seq datasets.^24^^,^^25^ VPS35 mRNA is broadly and variably expressed throughout the mouse brain across different cell types including multiple glial cells at low levels but with particular enrichment in neurons and oligodendrocytes or oligodendrocyte progenitor cells (Fig. 1A, Supplementary Table 1). Within distinct neuronal populations, VPS35 is broadly expressed with enrichment in serotonergic, cholinergic, noradrenergic and dorsal root ganglia populations (Fig. 1B, Supplementary Table 2). Neuronal VPS35 expression is distributed across multiple anatomic regions with particular enrichment in frontal cortical regions, hippocampus, striatum and cerebellum (Fig. 1C, Supplementary Table 3). Therefore, VPS35 transcripts are broadly expressed throughout the mouse brain with VPS35 likely playing important roles in many distinct cell types including neurons.

To explore the role of VPS35 in neurons, we first developed conditional *VPS35* knockout mice using gene-targeted ES cells. Mice were generated by injection of targeted ES cells [*VPS35^tm1a(EUCOMM)Hmgu^*] into the inner cell mass of C57BL/6N blastocysts to produce chimeric mice. Germline founders were subsequently identified through breeding and genomic PCR. Targeted mice harbour a KO-first allele containing a *loxP*-flanked exon 6 preceded by an *FRT*-flanked LacZ-Neo reporter cassette that disrupts expression from the *VPS35* locus (Fig. 1D). *VPS35* KO-first mice were next crossed with PGK1-FLPo transgenic mice to remove the LacZ-Neo cassette by FLPo-mediated *FRT* site recombination to generate cKO mice with a floxed exon 6 (Fig. 1D). Initially, KO-first ES cells [clone D11, *VPS35^tm1a(EUCOMM)Hmgu^*] were expanded and confirmed for correct gene-targeting by Southern blot analysis of restriction-digested genomic DNA using an LacZ DNA probe (Fig. 1D and E). The resulting *VPS35^+/^*^*−*^ KO-first mice were confirmed by PCR genotyping of tail genomic DNA using primer pairs flanking the 5ʹ or 3ʹ ends of the targeted cassette (Fig. 1D and F). Notably, intercrossing of *VPS35^+/^*^*−*^ KO-first mice fails to produce *VPS35*^*−*^^*/*^^*−*^ mice, implying embryonic lethality, thereby further confirming correct gene-targeting. Western blot analysis of hemi-brain extracts reveals reduced levels of VPS35 protein and a corresponding reduction of the retromer subunits, VPS26 and VPS29, in *VPS35^+/^*^*−*^ KO-first mice as expected, with normal levels of VPS35 protein restored by crossing *VPS35^+/^*^*−*^ KO-first and FLPo mice to generate *VPS35^flox/+^* cKO mice (Fig. 1G). *VPS35^flox/+^/*FLPo mice were subsequently crossed to C57BL/6N mice to remove the FLPo transgene, and then intercrossed to produce viable *VPS35^flox/flox^* mice at the expected frequency. *VPS35^flox/flox^* mice with pan-neuronal deletion were obtained by first crossing *VPS35^flox/+^* mice with Synapsin-1-Cre (Syn1-Cre) transgenic mice, and then crossing resulting *VPS35^flox/+^*/Syn1-Cre progeny with *VPS35^flox/flox^* mice to produce four genotypes at a frequency of ∼25% (Fig. 2A). Syn1-Cre mice express Cre recombinase from the rat synapsin-1 promoter in differentiated neuronal cells throughout the brain and spinal cord beginning at E12.5.^26^ For the remainder of this study, we compare mixed-sex cohorts of *VPS35^flox/flox^*/Syn1-Cre mice, with the broad neuronal-specific homozygous deletion of *VPS35*, to their *VPS35^flox/flox^* or *VPS35^flox/+^* littermates.

### Neuronal-specific *VPS35* conditional knockout mice display motor abnormalities and post-natal lethality

We first confirmed the neuronal distribution of Cre recombinase activity in Syn1-Cre mice by crossing with mT/mG reporter mice that express membrane-localized tdTomato before or GFP after Cre-mediated excision from the ROSA26 locus.^27^ In adult Syn1-Cre/mT/mG mice, Cre activity (GFP) is abundantly detected throughout the entire brain and spinal cord (Supplementary Fig. 1B) whereas in younger mice (at P10) Cre activity is restricted to the hippocampus, cerebellum, brainstem and most prominently the spinal cord (Fig. 2B, Supplementary Fig. 1B). GFP is not detected in mT/mG mice in the absence of Cre (Fig. 2B, Supplementary Fig. 1B). Cre activity is also detected in the spinal cord as early as P1 (Supplementary Fig. 1B). In *VPS35^flox/+^* and *VPS35^flox/flox^* mice, we confirm the excision of *VPS35* exon 6 in brain but not peripheral tissue (liver) only in the presence of Syn1-Cre by genomic PCR (Fig. 2C). Using RNAscope-fluorescence *in situ* hybridization to detect VPS35 mRNA in brain sections from P10 mice, we confirm decreased VPS35 mRNA expression in *VPS35^flox/flox^*/Syn1-Cre mice compared to Cre-negative control littermates, particularly in NeuN-positive neurons of the dentate gyrus and brainstem, confirming prominent Cre activity in these regions (Fig. 2D). In the spinal cord, VPS35 mRNA expression is detected in neurons with particular enrichment in ventral horn motor neurons of Cre-negative mice that is completely abolished in *VPS35^flox/flox^*/Syn1-Cre mice (Fig. 2D). VPS35 mRNA localization is confirmed in NeuN-positive neurons and choline acetyltransferase (ChAT)-positive motor neurons throughout the spinal cord of Cre-negative mice at P10 by combining RNAscope-fluorescence *in situ* hybridization and immunofluorescence (Fig. 2E), with the highest VPS35 fluorescence intensity detected in ChAT-positive neurons relative to NeuN-positive (ChAT-negative) neurons (Supplementary Fig. 1A). Importantly, quantitation of VPS35 fluorescent intensity reveals a near-complete loss of VPS35 signal in NeuN-positive and ChAT-positive the spinal cord neurons from *VPS35^flox/flox^*/Syn1-Cre mice compared to Cre-negative mice (Fig. 2E). These data confirm the neuronal-specific depletion of VPS35 mRNA in the spinal cord and other brain regions of *VPS35^flox/flox^*/Syn1-Cre mice, as expected (Fig. 2D and E). In addition, a marked yet non-significant reduction of VPS35 protein levels, as well as VPS26 and VPS29, is detected in spinal cord extracts from *VPS35^flox/flox^*/Syn1-Cre mice compared to control littermates by western blot (Fig. 2F). Notably, VPS35 and other subunits are not similarly reduced in hemi-brain extracts from *VPS35^flox/flox^*/Syn1-Cre mice at P12–P14 (Supplementary Fig. 1C), consistent with restricted Cre activity in the brain at this early age (see Fig. 2B, Supplementary Fig. 1B). A recent study reported that VPS35 knockout or knockdown in mammalian cells reduces the kinase activity of endogenous LRRK2, particularly towards its substrate Rab10.^28^ However, we detect normal phosphorylation levels of the LRRK2-specific substrates Rab10 (pThr73), Rab12 (pSer106) and Rab8a (pThr72 that also cross-reacts with pRab3a, pRab10, pRab35 and pRab43) in spinal cord and hemi-brain extracts from *VPS35^flox/flox^*/Syn1-Cre mice by Western blot (Supplementary Fig. 1D and E). These data confirm that *VPS35* cKO mice exhibit genomic deletion and reduced neuronal VPS35 expression in a Cre-dependent manner particularly in spinal cord, but lack evidence for reduced LRRK2 kinase activity in the CNS.

Mice produced from crosses of *VPS35^flox/+^*/Syn1-Cre and *VPS35^flox/flox^* mice are born at normal Mendelian frequencies, and appear healthy and unremarkable with no gross anatomical differences at P1 between genotypes (*VPS35^flox/+^*, *VPS35^flox/+^*/Syn1-Cre, *VPS35^flox/flox^* or *VPS35^flox/flox^*/Syn1-Cre; Fig. 2G). However, *VPS35^flox/flox^*/Syn1-Cre mice fail to reach weaning and exhibit post-natal lethality between P13 and P16 compared to other genotypes. During the post-natal period (P1–P15), these mice fail to develop normally with significantly reduced body weight and body length first detectable by P5 (Fig. 2H). At P10, *VPS35^flox/flox^*/Syn1-Cre mice first develop progressive hindlimb tremors and muscle weakness with worsening motor deficits leading to poor ambulation and righting reflexes that eventually culminate in limb paralysis and death (Fig. 2G, Supplementary Videos 1 and 2). Importantly, *VPS35^flox/flox^* mice lacking Cre or *VPS35^flox/+^*/Syn1-Cre mice expressing Cre, exhibit normal survival, motor activity, weight gain and body growth (Fig. 2G and H), indicating that neuronal Cre expression alone does not contribute to these phenotypes. These data demonstrate that the homozygous deletion of *VPS35* in neurons produces a progressive and fatal early-onset disease that resembles mouse models of familial ALS.

### Neuronal-specific *VPS35* conditional knockout mice display degeneration of spinal cord motor neurons

**Figure 3:**
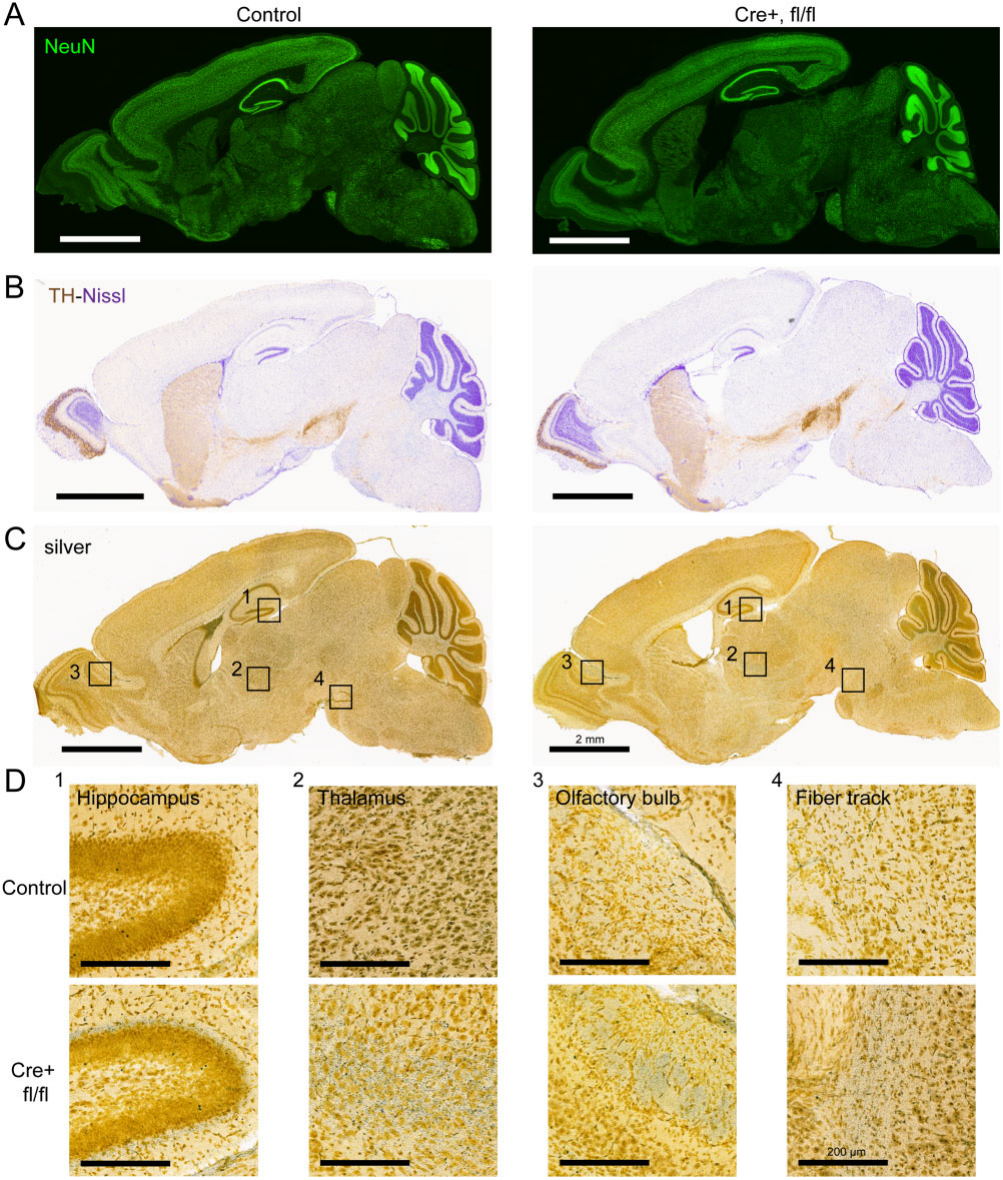
**Neuronal architecture and neurite damage in the brain of *VPS35* cKO mice crossed with Synapsin-1-Cre mice at P12–P14.** Representative sagittal brain sections from *VPS35^fl/fl^/Cre* or control (*VPS35^fl/fl^* or *VPS35^fl/wt^*) mice at P12 indicating (**A**) immunofluorescent labelling for the neuronal marker, NeuN or (**B**) immunohistochemistry with the dopaminergic neuronal marker, tyrosine hydroxylase (TH, brown) with Nissl staining (blue). (**C**) Images of Gallyas silver staining of sagittal brain sections indicating modest neurite degeneration and damage. (**D**) Representative high magnification images of Gallyas silver from the boxed regions shown in **C** revealing mild silver-positive neurites specifically in the hippocampus, thalamus, olfactory bulb and brainstem fibre tracts. Scale bars: 2 mm or 200 μm, as indicated.

Since familial *VPS35* mutations are linked to Parkinson’s disease and reduced VPS35 levels are associated with certain dementias,^11^^,^^12^^,^^17^^,^^18^ we initially focused on the impact of neuronal *VPS35* depletion in the brains of mice at P12. Notably, however, we observe no obvious differences in overall brain architecture or neuronal number, as suggested by immunofluorescence analysis of NeuN-positive neurons in *VPS35^flox/flox^*/Syn1-Cre mice compared to their control littermates lacking Cre (Fig. 3A). Consistent with this observation, cortical thickness or total hippocampal area, a specific region where VPS35 mRNA deletion is confirmed in this model (Fig. 2B), are normal in *VPS35^flox/flox^*/Syn1-Cre mice (Supplementary Fig. 2) indicating a lack of atrophy. Immunohistochemical analysis of tyrosine hydroxylase reveals a normal intact nigrostriatal dopaminergic pathway, whereas Nissl staining further confirms a lack of obvious cell loss or abnormal brain morphology (Fig. 3B). Gallyas silver staining fails to detect any major neurite degeneration throughout the brain, although modest silver-positive processes are observed selectively within the olfactory bulb, thalamus, hippocampus (dentate gyrus) and brainstem of *VPS35^flox/flox^*/Syn1-Cre mice at P10 supporting mild neurite damage or dysfunction (Fig. 3C and D).

**Figure 4:**
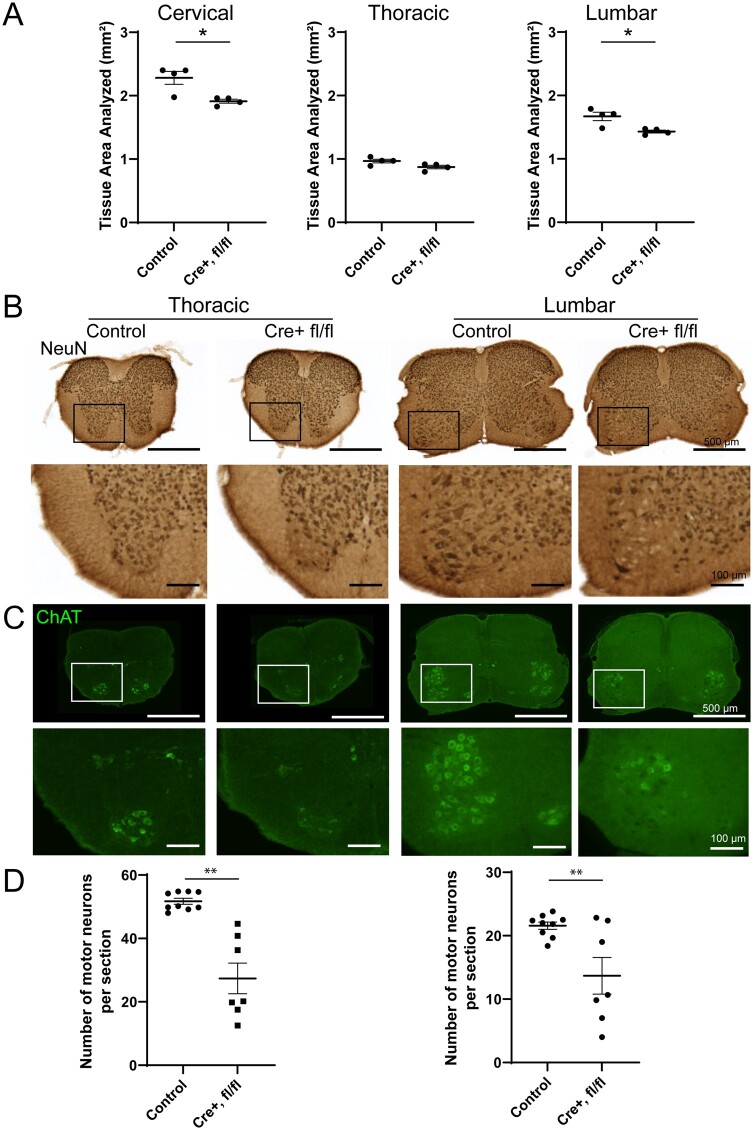
**Selective loss of ventral horn motor neurons in the spinal cord of *VPS35* cKO mice crossed with Synapsin-1-Cre mice at P12–P14.** (**A**) Quantitation of total area (mm^2^) of coronal sections from cervical, thoracic and lumbar spinal cord of *VPS35^fl/fl^/Cre* or control (*VPS35^fl/fl^* or *VPS35^fl/wt^*) mice. Bars represent the mean ± SEM (*n* = 4 mice/genotype) sampled across three to six sections per mouse. *P < 0.05 by unpaired, two-tailed Student’s *t*-test. (**B, C**) Coronal sections of thoracic and lumbar spinal cord labelled by immunohistochemistry for NeuN (**B**), or by immunofluorescence for ChAT specifically labelling motor neurons (**C**) with high magnification images of the boxed regions shown at the bottom of each panel. NeuN and ChAT immunoreactivity reveal the loss of large motor neurons in the ventral horns of *VPS35^fl/fl^/Cre* mice. (**D**) Graphs indicating the number of ChAT-positive motor neurons in the ventral horns per coronal section (35 µm-thick) from thoracic (*left*) or lumbar (*right*) regions. Bars represent the mean ± SEM for *VPS35^fl/fl^/Cre* mice (*n* = 7 mice) and control mice (*VPS35^fl/fl^*, *VPS35^fl/wt^*, *VPS35^wtlwt^*; *n =* 9 mice). **P < 0.01 by unpaired, two-tailed Student’s *t*-test. Scale bars: 100 or 500 μm, as indicated.

**Figure 5:**
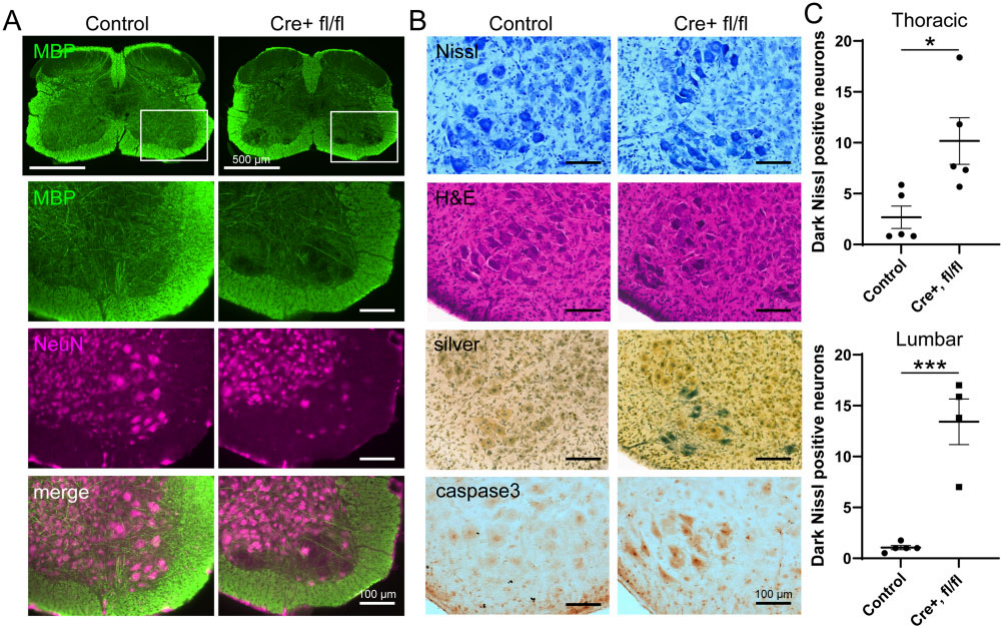
**Degeneration of ventral horn motor neurons in the spinal cord of *VPS35* cKO mice crossed with Synapsin-1-Cre mice at P12.** (**A**) Immunofluorescence co-localization of myelin basic protein (MBP) and NeuN in the lumbar spinal cord of *VPS35^fl/fl^/Cre* or control (*VPS35^fl/fl^* or *VPS35^fl/wt^*) mice. Representative high magnification images are shown from the boxed region of the ventral horn (*upper panel*). The loss of large NeuN-positive motor neurons and associated MBP-positive fibre tracts in *VPS35^fl/fl^/Cre* mice is indicated. (**B**) Representative histological staining of lumbar spinal cord (ventral horn) sections with cresyl violet (Nissl) and haematoxylin/eosin, in addition to Gallyas silver stain or immunohistochemical labelling of activated caspase-3, from *VPS35^fl/fl^/Cre* or control (*VPS35^fl/fl^* or *VPS35^fl/wt^*) mice. Notice that large ventral horn motor neurons display condensed dark staining for Nissl and haematoxylin/eosin with abnormal morphology, and stain positive for silver and activated caspase-3 in *VPS35^fl/fl^/Cre* mice. (**C**) Quantitative counts of dark, condensed Nissl-positive neurons in thoracic and lumbar spinal cord sections from *VPS35^fl/fl^/Cre* mice (*n* = 5 mice) and control mice (*VPS35^fl/fl^*, *VPS35^fl/wt^*, *VPS35^wtlwt^*; *n =* 5 mice) sampled across 5–6 sections per animal. Bars indicate the mean ± SEM. *P < 0.05 or ***P < 0.001 by unpaired, two-tailed Student’s *t*-test. Scale bars: 100 or 500 μm, as indicated.

Given the lack of obvious neurodegeneration within the brain, we focused instead on the spinal cord due to the progressive limb paralysis and early lethality observed in *VPS35^flox/flox^*/Syn1-Cre mice as well as the prominent Cre activity in this region of Syn1-Cre mice at P10 (Fig. 2, Supplementary Fig. 1). Anatomically, the spinal cord of *VPS35^flox/flox^*/Syn1-Cre mice at P12–P14 reveals quantitative evidence of modest atrophy compared to control mice, with a significant reduction of total tissue area in cervical and lumbar sections (Fig. 4A). Intriguingly, an obvious loss of large NeuN-positive motor neurons is clearly evident within the ventral horns of the thoracic and lumbar spinal cord of *VPS35^flox/flox^*/Syn1-Cre mice, whereas other non-motor neuronal populations appear relatively normal in number and morphology (Fig. 4B). Motor neurons were specifically labelled using ChAT antibody to reveal their selective loss in the ventral horn of P12–P14 mice (Fig. 4C). Systematic unbiased cell counting reveals a significant 40–50% loss of ChAT-positive motor neurons in the thoracic and lumbar spinal cord of *VPS35^flox/flox^*/Syn1-Cre mice compared to control littermates (Fig. 4D). Neuronal loss is accompanied by a specific loss of myelination restricted to grey matter regions of the ventral horn as indicated by immunofluorescent co-labelling of myelin basic protein and NeuN (Fig. 5A). Importantly, no loss of ChAT-positive motor neurons is detected in *VPS35^flox/flox^*/Syn1-Cre mice at P5, suggesting that neurodegeneration occurs in a progressive manner (Supplementary Fig. 3A). To confirm the loss of motor neurons at P12, Nissl (cresyl violet) and haematoxylin/eosin staining of spinal cord sections was conducted. Surprisingly, however, ventral horn motor neurons are still present and normal in number yet many appear morphologically abnormal or damaged, including evidence for obvious neuronal atrophy and condensed dark pyknotic staining (Fig. 5B). Quantitation of the number of dark pyknotic Nissl-positive motor neurons reveals a significant increase in the thoracic and lumbar spinal cord of *VPS35^flox/flox^*/Syn1-Cre mice compared to control littermates at P12 (Fig. 5C). Motor neurons in the ventral horn of P12 mice also selectively reveal dense staining for Gallyas silver (Fig. 5B), which are absent from mice at P5 (Supplementary Fig. 3B), suggesting that motor neurons are undergoing progressive degeneration. Furthermore, ventral horn motor neurons also contain nuclei that stain positive for activated, cleaved caspase-3 (Asp175) by immunohistochemistry, indicating that motor neurons are actively undergoing apoptosis (Fig. 5B). Importantly, non-motor neuronal populations throughout the spinal cord do not exhibit abnormal morphology or number, and do not stain positive for Gallyas silver or cleaved caspase-3 (Figs 4 and 5), indicating that the degeneration of ventral horn motor neurons is relatively selective within the spinal cord, consistent with highest VPS35 expression in this neuronal population (see Fig. 2D and E). Collectively, these data suggest that *VPS35^flox/flox^*/Syn1-Cre mice develop a progressive and fatal disease characterized by motor abnormalities that are associated with the progressive and selective degeneration of spinal cord motor neurons.

### Neuronal *VPS35* deletion induces protein inclusions in the spinal cord

**Figure 6:**
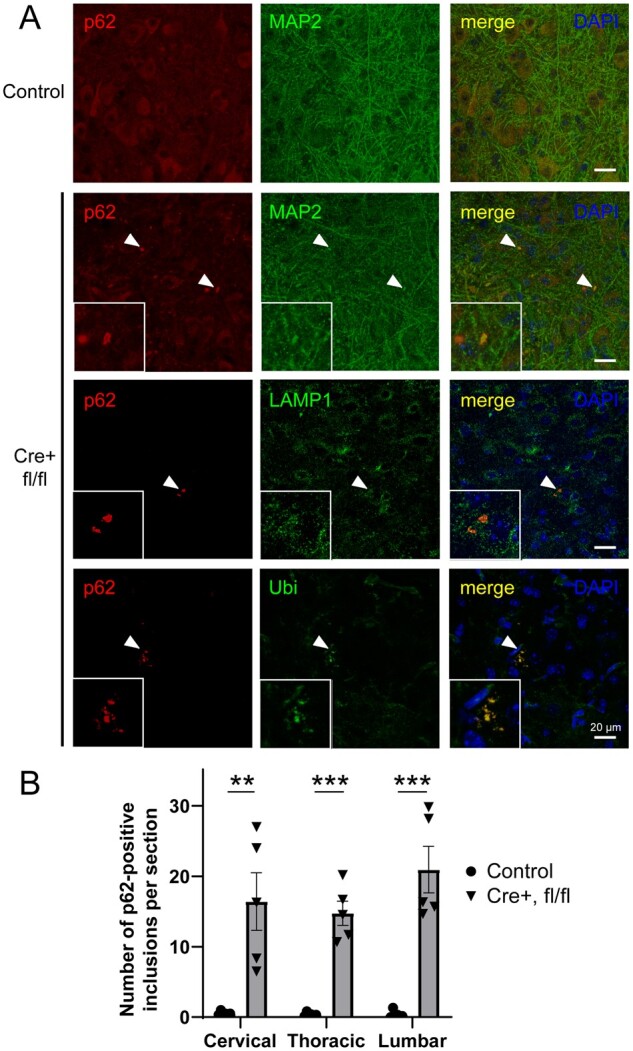
**Accumulation of protein inclusions throughout the spinal cord grey matter of *VPS35* cKO mice crossed with Synapsin-1-Cre mice at P12–P14.** (**A**) Representative immunofluorescent confocal co-localization in spinal cord coronal sections from *VPS35^fl/fl^/Cre* (*n* = 7 mice) or control (*VPS35^fl/fl^*, *VPS35^fl/wt^* or *VPS35^wt/wt^/Cre*; *n* = 9 mice) mice for p62/SQSTM1 (red) together with the neuronal marker microtubule-associated protein 2, lysosomal marker LAMP1 or ubiquitin (Ubi) (green). DAPI nuclear stain is shown in merged images. Arrowheads indicate p62-positive inclusions with high magnification images shown in the boxed regions (lower left). (**B**) Quantitative counts of p62-positive inclusions in cervical, thoracic and lumbar spinal cord sections of *VPS35^fl/fl^/Cre* mice (*n* = 5 mice) and control mice (*VPS35^fl/fl^*, *VPS35^fl/wt^*, *VPS35^wtlwt^*; *n =* 6 mice) sampled across six sections per animal. Bars indicate the mean ± SEM. **P < 0.01 or ***P < 0.001 by unpaired, two-tailed Student’s *t*-test. Scale bars: 20 μm.

**Figure 7:**
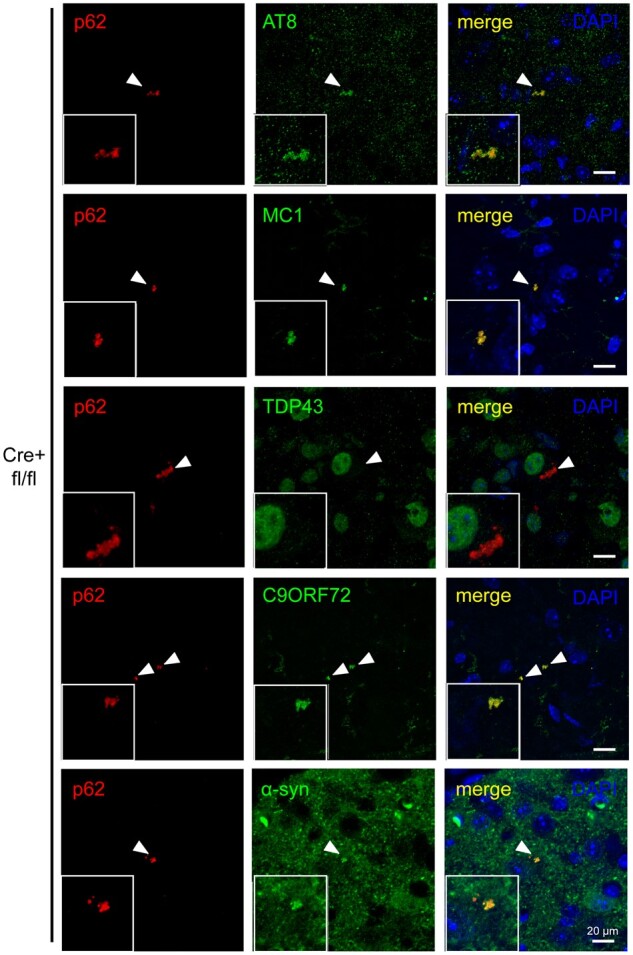
**Neurodegenerative protein markers co-localizing with p62-positive inclusions in the spinal cord grey matter of *VPS35* cKO mice crossed with Synapsin-1-Cre mice at P12–P14.** Representative immunofluorescent confocal co-localization in coronal sections of spinal cord from *VPS35^fl/fl^/Cre* mice for p62/SQSTM1 (red) and phosphorylated tau (pSer202/pThr205; AT8), abnormal conformation-specific tau (MC1), TDP-43, C9ORF72 or α-synuclein (all green) with nuclear DAPI (blue). Arrowheads indicate p62-positive inclusions with high magnification images shown in the boxed regions (lower left). Scale bars: 20 μm.

*VPS35* depletion in neurons has been linked to protein aggregation or the accumulation of α-synuclein in mice.^13^^,^^23^ To detect protein aggregation in neuronal-specific *VPS35* cKO mice, immunofluorescent labelling of brain and spinal cord sections was conducted for the ubiquitin-binding protein, p62/SQSTM1, that plays a role in selective autophagy and often co-labels protein aggregates. Interestingly, large intracellular p62-positive inclusions are sparsely distributed within grey matter of the cervical, thoracic and lumbar spinal cord of *VPS35^flox/flox^*/Syn1-Cre mice at P12 relative to control littermates (Fig. 6A) but are generally absent from the brain. Quantitative analysis reveals a marked and significant increase in p62-positive inclusions throughout all levels of the spinal cord of *VPS35^flox/flox^*/Syn1-Cre mice (Fig. 6B). These p62-positive inclusions are observed in both the ventral and dorsal horns of the spinal cord, are not specific to motor neurons (Fig. 6), and notably also appear early in mice at P5 (Supplementary Fig. 3C). Confocal co-localization studies indicate that p62-positive inclusions co-localize with the neuronal marker, microtubule-associated protein 2, suggesting that inclusions are neuronal and mostly localized within processes (Fig. 6A). Importantly, p62-positive inclusions co-localize with ubiquitin, which confirms the specificity of p62 labelling and suggests that these inclusions may also contain ubiquitinated proteins. The p62-positive inclusions also co-localize with the lysosomal marker, LAMP1 (Fig. 6A), suggesting the accumulation of lysosomal structures that potentially contain proteins undergoing degradation. Intriguingly, p62-positive inclusions also co-localize with a number of endogenous neurodegenerative protein markers in the spinal cord, including abnormal phosphorylated (pSer202/pThr205; clone AT8) or conformation-specific (clone MC1) forms of tau, C9ORF72 and α-synuclein (total; clone Syn1) but not with TDP-43 (Fig. 7). These data suggest that neuronal p62-positive protein inclusions characterized by autophagy-lysosomal and protein aggregation markers are induced early and broadly in the spinal cord of *VPS35^flox/flox^*/Syn1-Cre mice prior to motor symptom-onset or obvious neurodegeneration.

### Neuronal-specific *VPS35* deletion induces early reactive gliosis in the spinal cord

**Figure 8:**
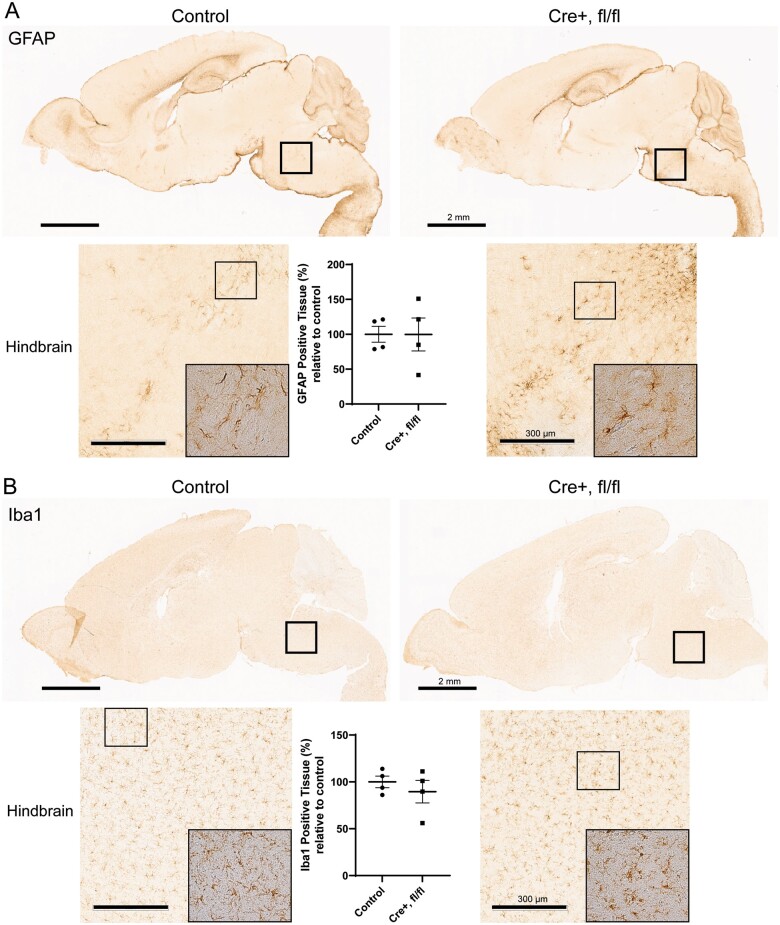
**Absence of astrogliosis or microglial proliferation in the brain of *VPS35* cKO mice crossed with Synapsin-1-Cre mice.** Immunohistochemical analysis of sagittal brain sections from *VPS35^fl/fl^/Cre* or control (*VPS35^fl/f^* or *VPS35^fl/wt^*) mice at P12–P14 for **A** the astrocyte marker, GFAP, or **B** the microglial marker, Iba1. High magnification images of the hindbrain/brainstem (lower panels) taken from the boxed region of each sagittal section (upper panel) are shown. Lower panels also contain high magnification images (boxed region) revealing individual glial cells. (**A, B**) Graphs indicate quantitation of per cent area positive for GFAP or Iba1 immunoreactivity (expressed relative to control mice) in the hindbrain for *VPS35^fl/fl^/Cre* mice (*n* = 4 mice) and control mice (*VPS35^fl/fl^, VPS35^fl/wt^; n =* 4 mice) using HALO analysis software. Bars indicate the mean ± SEM. No significant differences are found by unpaired, two-tailed Student’s *t*-test. Scale bars: 2 mm or 300 μm, as indicated.

**Figure 9:**
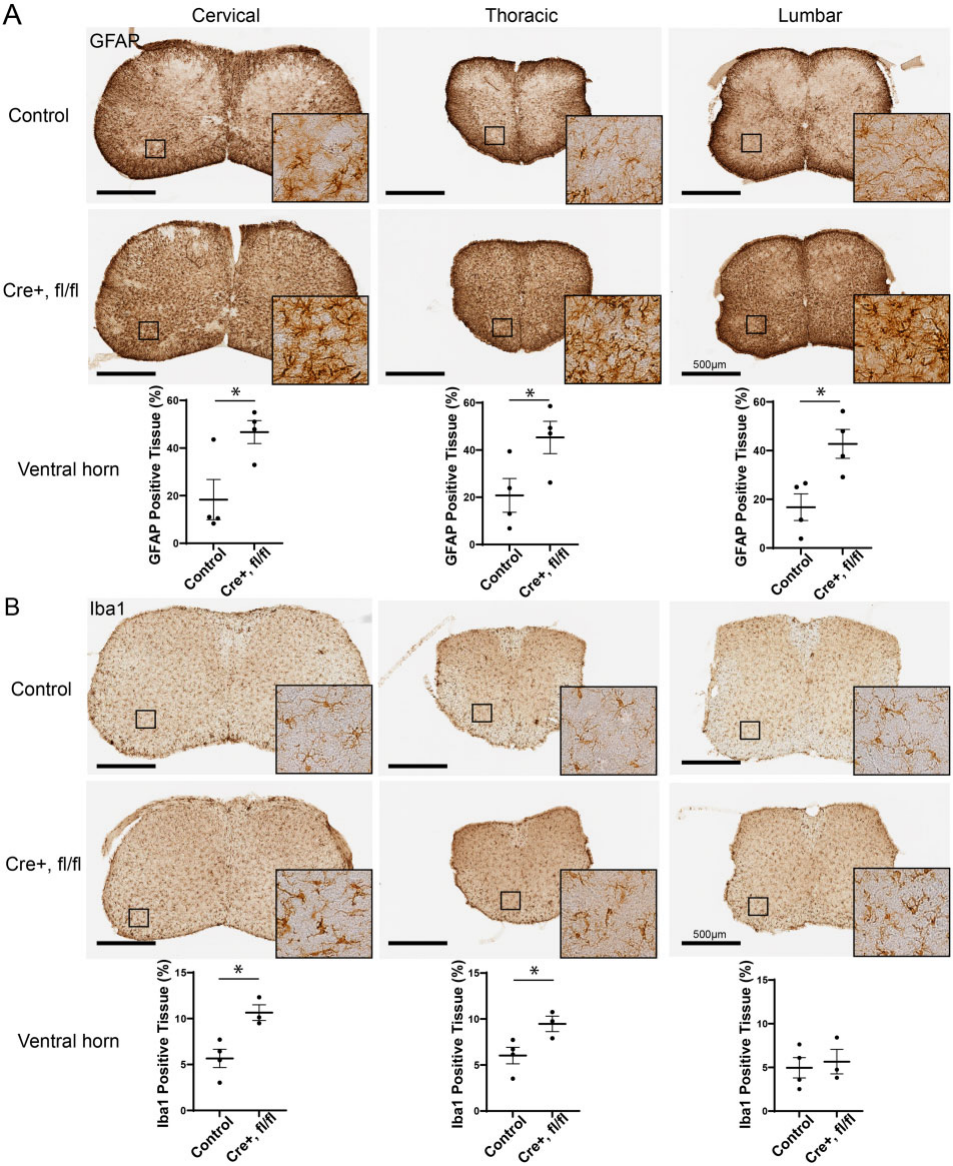
**Pronounced reactive gliosis in the spinal cord of *VPS35* cKO mice crossed with Synapsin-1-Cre mice at P12–P14.** (**A, B**) Immunohistochemical analysis of cervical, thoracic and lumbar spinal cord from *VPS35^fl/fl^/Cre* (*n* = 4 mice) or control (*VPS35^fl/fl^*, *VPS35^fl/wt^* or *VPS35^wt/wt^/Cre*; *n* = 4 mice) mice for the astrocyte marker, GFAP (**A**) or the microglial marker, Iba1 (**B**). High magnification images of individual glial cells within the spinal cord ventral horn are shown from the boxed regions (left), as indicated. (**A, B**) Graphs indicate quantitation of GFAP or Iba1 immunoreactivity within the ventral horn grey matter for each spinal cord region using HALO analysis software. Bars represent the mean ± SEM (*n* = 4 mice/genotype) sampled across three to six sections per animal, with GFAP- or Iba1-positive immunoreactivity expressed as a per cent of total tissue area analysed. *P < 0.05 by unpaired, two-tailed Student’s *t*-test. Scale bars: 500 μm.

To further characterize the neuropathology of *VPS35^flox/flox^*/Syn1-Cre mice, we sought to evaluate signs of neuroinflammation that may result from neuronal *VPS35* depletion and/or from neurodegenerative processes. At P12, immunohistochemistry and quantitative analysis for the astrocyte marker, GFAP, does not reveal evidence for reactive astrogliosis within the brains of *VPS35^flox/flox^*/Syn1-Cre mice compared to control littermates (Fig. 8A), including within the hindbrain region where Cre activity is highest at this age (Fig. 2B). Similarly, no obvious alterations in the number or morphology of Iba1-positive microglia are evident in the brains of these mice by quantitative immunohistochemistry (Fig. 8B). In contrast, robust reactive astrogliosis is detected throughout the grey matter of the spinal cord of *VPS35^flox/flox^*/Syn1-Cre mice at P12 (Fig. 9A), with modest astrogliosis already observed by P5 (Supplementary Fig. 3D). Quantitation of GFAP-positive labelling in the ventral horns reveals a significant marked increase in GFAP-positive area in the cervical, thoracic and lumbar spinal cord of *VPS35^flox/flox^*/Syn1-Cre mice compared to control littermates (Fig. 9A, Supplementary Fig. 4). Iba1-positive microglial labelling is also significantly increased in the ventral horn of cervical and thoracic spinal cord from *VPS35^flox/flox^*/Syn1-Cre mice at P12, indicating robust microgliosis, yet surprisingly not within the lumbar spinal cord (Fig. 9B, Supplementary Fig. 4). Taken together, these data suggest that depletion of *VPS35* in neurons induces widespread reactive gliosis throughout the spinal cord prior to the onset of motor neuron degeneration.

## Discussion

Here, we report the development of conditional *VPS35* knockout mice harbouring a floxed exon 6, with neuronal-specific *VPS35* deletion induced by crossing to synapsin-1-Cre transgenic mice. Although mice were grossly normal at birth and produced at normal Mendelian frequencies, neuronal *VPS35* deletion caused a rapid and progressive neurological disease over ∼15 days characterized by motor deficits, impaired growth and early post-natal lethality. Surprisingly, *VPS35* knockout mice at end-stage disease exhibit normal brain architecture, morphology and neuronal number, with only modest signs of silver-positive axonal damage in discrete neuronal terminal fields. Instead, we find that *VPS35* knockout mice exhibit the relatively selective and robust degeneration of motor neurons throughout the spinal cord ventral horns, characterized by a pronounced loss of NeuN-positive and ChAT-positive immunolabelling, abnormal neuronal atrophy and morphology, and degenerating neuronal soma that stain positive for Gallyas silver and activated caspase-3. We also find evidence that neuronal-specific *VPS35* deletion induces the early formation of protein inclusions throughout neuronal populations within the spinal cord, that co-label for p62, ubiquitin, LAMP1, abnormal tau, C9ORF72 and α-synuclein, suggesting abnormal protein accumulation and/or endolysosomal function. Accompanying the robust neuronal degeneration in the spinal cord of *VPS35* knockout mice, we find evidence for early and robust neuroinflammation in grey matter regions throughout the spinal cord, including reactive astrocytes and increased microglia, prior to the onset of neuronal damage. Collectively, our data demonstrate that one of the earliest effects of pan-neuronal *VPS35* deletion in mice is motor neuron degeneration and impaired survival that closely resembles rodent models of familial ALS. Our study suggests that ventral horn motor neurons critically require VPS35 during early post-natal development for their normal maintenance and survival, and supports an emerging role for VPS35 and the retromer in the pathophysiology of ALS.

The selective impact of *VPS35* deletion on ventral horn motor neurons of the spinal cord most likely results from selective expression of Cre recombinase from the synapsin-1 promoter during post-natal development. While the synapsin-1 promoter typically drives widespread neuronal expression in adult mice and is therefore considered pan-neuronal,^26^ Cre recombinase activity is largely restricted to the brainstem and spinal cord during the early post-natal period (P1–P10) as indicated using mT/mG reporter mice (see Fig. 2B). These data are further supported by RNAscope analysis that reveals (i) prominent VPS35 mRNA expression in motor neurons and (ii) a complete loss of VPS35 mRNA in the spinal cord in Cre-positive animals (Fig. 2D and E). At the protein level, we detect reduced VPS35 and retromer subunits in the spinal cord by P12, compared to normal retromer levels in the whole brain, and robust evidence of a relatively selective effect of *VPS35* deletion on motor neuron survival. While we do not find evidence for the loss of additional neuronal populations in the spinal cord, we do observe broader effects of *VPS35* deletion including neuronal p62-positive inclusions and reactive gliosis in grey matter regions. Therefore, it is likely that neuronal *VPS35* deletion impacts other non-motor neurons directly in addition to glial cells indirectly throughout the spinal cord, yet without impacting their survival during the post-natal period (≤P15) we assessed. Our study demonstrates that spinal cord motor neuron survival is particularly dependent on normal retromer function during early post-natal development and consistent with this finding, VPS35 mRNA levels are highest in motor neurons relative to other neuronal populations of the spinal cord at this age (Fig. 2E, Supplementary Fig. 1A). However, it is likely that many distinct neuronal populations are vulnerable to the loss of VPS35. For example, the selective deletion of *VPS35* in embryonic pyramidal neurons in the mouse cortex using Neuro6d-Cre produces similar phenotypes to those observed here in motor neurons, with the formation of p62- and LC3-II-positive inclusions, the accumulation of ubiquitinated proteins, activated caspase-3 and selective neuronal degeneration by P21 accompanied by early reactive astrogliosis and microglial activation.^23^ Similarly, selective *VPS35* deletion in dopaminergic neurons using DAT-Cre induces their robust degeneration in the substantia nigra by 2–3 months of age.^14^ These conditional KO mouse studies support observations in human brains from Alzheimer’s disease, different tauopathies and ALS subjects demonstrating reduced levels of retromer subunits in affected brain regions or neuronal populations.^17^^,^^18^^,^^21^ Our future studies will further explore the selective neuronal vulnerability to the loss of retromer function via the inducible pan-neuronal deletion of *VPS35* in adult mice following post-natal development.

The early vulnerability of spinal cord motor neurons to the loss of *VPS35* expression potentially supports an unappreciated role for the retromer in the pathophysiology of ALS. Neuronal *VPS35* deletion recreates some of the hallmark phenotypes observed in mouse models of familial ALS based upon *SOD1* mutations, including the selective loss of motor neurons, reactive astrogliosis, motor deficits and limb paralysis, and premature survival.^29^^,^^30^ While reduced retromer levels have long been shown in Alzheimer’s disease brains,^17^ and *VPS35* mutations linked with familial Parkinson’s disease,^11^^,^^12^^,^^31^ a connection between ALS and the retromer has only recently emerged. A very recent study reports that endogenous VPS35 protein levels and other retromer subunits are selectively reduced in spinal cord motor neurons in ALS-linked G93A SOD1 mice, as well as in ventral horn motor neurons from sporadic ALS brains and iPSC-derived motor neurons derived from ALS subjects bearing *SOD1* mutations.^21^ Intriguingly, pharmacological retromer stabilization using a novel small molecule chaperone based upon R55 that binds at the C-terminal VPS35-VPS29 interface, was shown to increase VPS35 levels in mouse brain and attenuate neurodegenerative phenotypes in G93A SOD1 mice.^21^ Retromer stabilization reversed Golgi fragmentation and degeneration of motor neurons, reduced sciatic nerve degeneration, restored the levels of retromer cargo, and reduced the accumulation of ubiquitinated proteins in G93A SOD1 mice.^21^ These data suggest that the spontaneous age-dependent reduction of retromer subunits may contribute to the degeneration of motor neurons in ALS, albeit through an unknown mechanism, with retromer stabilization proving beneficial in rodent models. Our study further extends and validates this work by oppositely demonstrating that the selective deletion of *VPS35* in ventral horn motor neurons is sufficient alone to elicit their rapid degeneration that precipitates motor deficits and impaired survival similar to ALS. Our *VPS35* KO model displays an accelerated and more aggressive neurological phenotype compared to G93A SOD1 mice,^30^ most likely reflecting the consequences of a complete loss of VPS35 in motor neurons in our model relative to reduced levels in the SOD1 mice which survive into adulthood.

The exact mechanism by which motor neurons degenerate due to VPS35 loss is unclear. However, we and other studies find evidence for abnormal endolysosomal pathway function, including the accumulation of p62- and LC3-II-positive inclusions that contain ubiquitin, alterations in lysosomal markers, and silver-positive degenerating neuronal soma most likely containing argyrophilic lysosomes, suggesting that lysosomal activity is potentially perturbed.^23^ In addition, degenerating motor neurons (or cortical pyramidal neurons^23^) exhibit signs of atrophy, abnormal morphology and pyknosis as well as DNA damage and activated caspase-3, indicating that these neurons undergo apoptosis. A recent study in cortical pyramidal neurons revealed that *VPS35* deletion induces the abnormal lysosomal accumulation of the retromer cargo sortilin-1, with knockdown of sortilin-1 attenuating many of the deficits in *VPS35* cKO mice.^23^ Furthermore, sortilin-1 overexpression alone in primary cortical neurons was sufficient to increase LAMP1 puncta size, reduce lysosomal acidity, and induce p62-positive inclusions,^23^ suggesting that sortilin-1 accumulation may mediate the lysosomal deficits induced by *VPS35* deletion. In addition to sortilin-1 sorting, the loss of VPS35 expression has been proposed to induce neuronal degeneration in mouse models via at least two distinct mechanisms. *VPS35* deficiency was shown to (i) impair mitochondrial fusion and function via an increase in the mitochondrial E3 ubiquitin ligase MUL1 leading to enhanced degradation of mitofusin 2^14^ and (ii) impair the endosomal retrieval and accelerate the degradation of LAMP2A^13^, a receptor for chaperone-mediated autophagy. At this juncture, it is unclear whether sortilin-1 or other retromer cargo plays a similar role in motor neurons lacking VPS35. Future studies will explore the role of retromer cargo and lysosomal function in mediating the effects of *VPS35* deletion in motor neurons.

While retromer levels are reduced in dementias and ALS,^17^^,^^18^^,^^21^ it is unclear whether retromer subunits are similarly reduced in Parkinson’s disease brains due to conflicting reports.^32^^,^^33^ Instead, *VPS35* mutations are linked to late-onset familial Parkinson’s disease via a single heterozygous mutation (D620N),^31^ whereas *VPS35* mutations have not yet been identified in familial forms of Alzheimer’s disease, frontotemporal dementias or ALS. It has been proposed that the D620N mutation manifests disease via a loss-of-function mechanism based largely on studies in heterozygous or conditional *VPS35* KO mice that have normal survival yet develop substantia nigra dopaminergic neuronal loss.^13^^,^^14^ However, homozygous *D620N VPS35* knock-in mice display normal survival compared to homozygous *VPS35* KO that die during early embryonic development, whereas compound heterozygotes (*VPS35^D620N/^*^*−*^) also display a normal lifespan indicating that a single *D620N* allele is sufficient for survival.^15^ If the D620N mutation acts via a loss-of-function mechanism then one would predict that homozygous *D620N VPS35* mice, or compound heterozygotes (*VPS35^D620N/^*^*−*^), would phenocopy the *VPS35* cKO/Syn-1-Cre mice described here, with motor deficits and early post-natal lethality. As this is not the case, it is likely that the D620N mutation acts via a gain-of-function or partial dominant-negative mechanism to cause Parkinson’s disease. While impaired survival and early motor deficits are not evident or robust in heterozygous *VPS35* KO mice or homozygous *D620N VPS35* mice,^13^^,^^15^^,^^16^^,^^34^ it is still possible that both models could develop more subtle motor neuron abnormalities or pathology and this will be of interest to explore in future studies given the early vulnerability of this neuronal population. By comparing the impact of neuronal deletion of *VPS35* in mice to existing *VPS35* mouse models, our data support the concept that Parkinson’s disease-linked *VPS35* mutations do not manifest a complete loss-of-function effect and instead may operate in a more subtle manner to alter retromer function.

Collectively, our study reveals a surprising and unappreciated vulnerability of ventral horn motor neurons of the spinal cord to the loss of VPS35 expression in mice during the early post-natal period. Our data reinforce the concept that motor neurons critically require normal retromer function for their maintenance and survival, and support a potential role for the retromer in the pathophysiology of ALS. Together with other studies,^17^^,^^18^^,^^21^ our data provide support for the use of pharmacological retromer stabilization as a potential therapeutic strategy for ALS as well as Alzheimer’s disease and frontotemporal dementias that display evidence of reduced retromer levels in affected neurons.

## Supplementary material

Supplementary material is available at *Brain Communications* online.

## Supplementary Material

fcab208_Supplementary_Data

## Abbreviations

ALS: amyotrophic lateral sclerosis
ChAT: choline acetyl transferase
cKO: conditional knockout
DAT: dopamine transporter
D620N: Asp620Asn
ES: embryonic stem
GFAP: glial fibrillary acidic protein
GFP: green fluorescent protein
Iba1: ionized calcium-binding adaptor molecule 1
LAMP1: lysosomal-associated membrane protein 1
NeuN: neuronal nuclear protein
SOD1: superoxide dismutase 1
SQSTM1: Sequestosome-1
TBS: Tris-buffered saline
VPS35: Vacuolar Protein Sorting 35 Orthologue
