# Supplementary material for: Neuronal VPS35 deletion induces spinal cord motor neuron degeneration and early post-natal lethality
Source: Brain Commun. 2021 Sep 10;3(3):fcab208. doi: 10.1093/braincomms/fcab208 (PMC8445400; doi:10.1093/braincomms/fcab208)

Supplementary Data

Supplementary Figures:

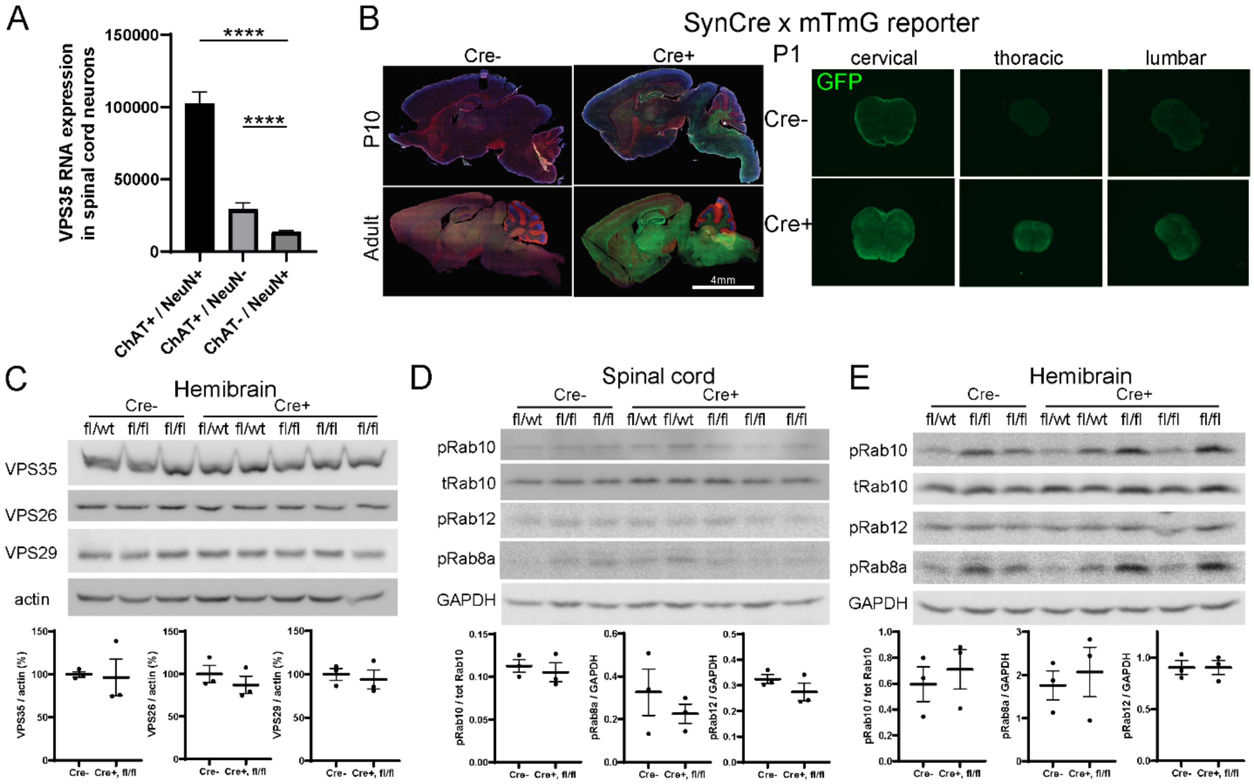

Figure S1. VPS35 mRNA is highly expressed in motor neurons of the spinal cord from Cre-negative mice, whereas retromer levels and LRRK2 activity remain normal in the brain of *VPS35* cKO mice crossed with Synapsin-1-Cre mice. (**A**) Levels of VPS35 mRNA detected by RNAscope in ChAT+/NeuN+ (*n* = 40), ChAT+/NeuN- (*n* = 12), ChAT-/NeuN- (*n* = 79) neurons from lumbar spinal cord sections of Cre-negative mice by confocal microscopy. Fluorescence intensity was measured using ImageJ and graph indicates corrected total cell fluorescence (CTCF) values for VPS35 signal in these neuronal populations. Bars indicate the mean ± SEM. ****P<0.0001 by unpaired, two-tailed Student's *t*-test as indicated. (**B**) Fluorescent images indicating the conversion of tdTomato (red) to GFP (green) after Cre-mediated recombination in sagittal brain sections (*left panels*) of mT/mG reporter mice crossed with Syn1-Cre mice at P10 or adult ages. Nuclear DAPI (blue). Coronal sections from cervical, thoracic and lumbar spinal cord of P1 mice from the same mouse crosses revealing GFP detection after Cre-mediated recombination (*right panels*). (**C**) Western blot analyses of soluble hemi-brain extracts derived from $VPS35^{fl/fl}/Cre$ or $VPS35^{fl/wt}/Cre$ mice compared to Cre-negative control mice ($VPS35^{fl/fl}$ or

VPS35$^{fl/wt}$) at P12-14 for VPS35, VPS26, VPS29 or actin. Graphs indicate densitometric quantitation of VPS35, VPS26 or VPS29 protein levels normalized to actin in *VPS35$^{fl/fl}$/Cre* (*n* = 3 mice) compared to control mice (*VPS35$^{fl/fl}$* or *VPS35$^{fl/wt}$*; *n* = 3 mice). (**D-E**) Western blot analyses of soluble spinal cord or hemi-brain extracts derived from the same mice as in (**C**) probed for LRRK2-specific phosphorylated forms of Rab10 (pThr73), Rab8a (pThr72) and Rab12 (pSer106) as well as total Rab10 and GAPDH. Graphs indicate densitometric quantitation of pRab10/total Rab10, pRab8a/GAPDH, pRab12/GAPDH in *VPS35$^{fl/fl}$/Cre* (*n* = 3 mice) compared to control mice (*VPS35$^{fl/fl}$* or *VPS35$^{fl/wt}$*; *n* = 3 mice). Bars represent mean ± SEM expressed as a percent of Cre-negative control mice. $P>0.05$ by unpaired, two-tailed Student's *t*-test.

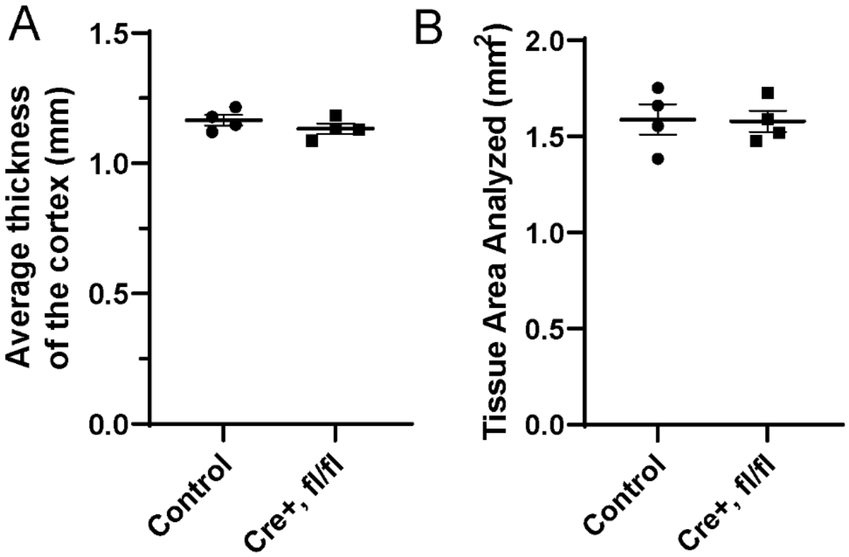

Figure S2. Atrophy is not detected in the brain of VPS35 cKO mice crossed with Synapsin-1-Cre mice at P12-14. (**A**) Cortical thickness (in mm; average taken of somatomotor area, somatosensory area and visual area) was measured using HALO analysis software on sagittal brain sections stained with Nissl from *VPS35$^{fl/fl}$/Cre* (*n* = 4 mice) compared to control mice (*VPS35$^{fl/fl}$* or *VPS35$^{fl/wt}$*; *n* = 4 mice). (**B**) Hippocampal area (in mm^2) measured using HALO software in sagittal brain sections from the same mice stained with Nissl. Bars represent mean ± SEM expressed in mm or mm^2. P>0.05 by unpaired, two-tailed Student's *t*-test.

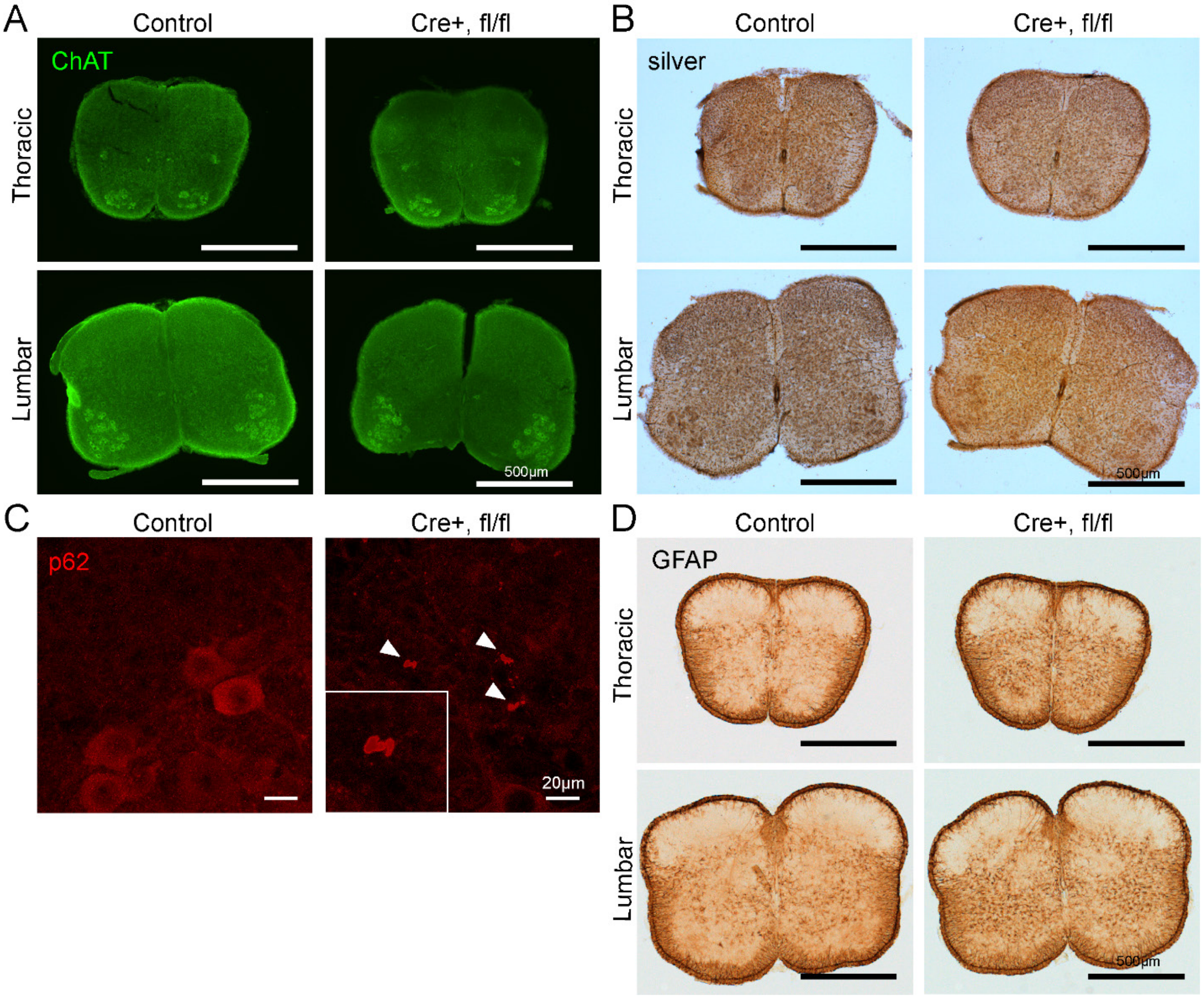

Figure S3. Early accumulation of protein inclusions and reactive astrogliosis in spinal cord of *VPS35* cKO mice crossed with Synapsin-1-Cre mice at P5. Immunohistochemical or histological analyses of thoracic and lumbar spinal cord from *VPS35*$^{fl/fl}$*/Cre* mice compared to control mice (*VPS35*$^{fl/fl}$) at an early age (P5) for (**A**) the motor neuron marker, choline acetyltransferase (ChAT), (**B**) Gallyas silver stain, (**C**) p62/SQSTM1 (arrowheads indicate p62-positive inclusions), and (**D**) the astrocyte marker, GFAP. Notice, motor neurons in the ventral horns exhibit normal number and lack silver positivity, yet spinal cord gray matter exhibits p62-positive inclusions and marked reactive astrogliosis. Scale bars: 20 or 500 μm, as indicated.

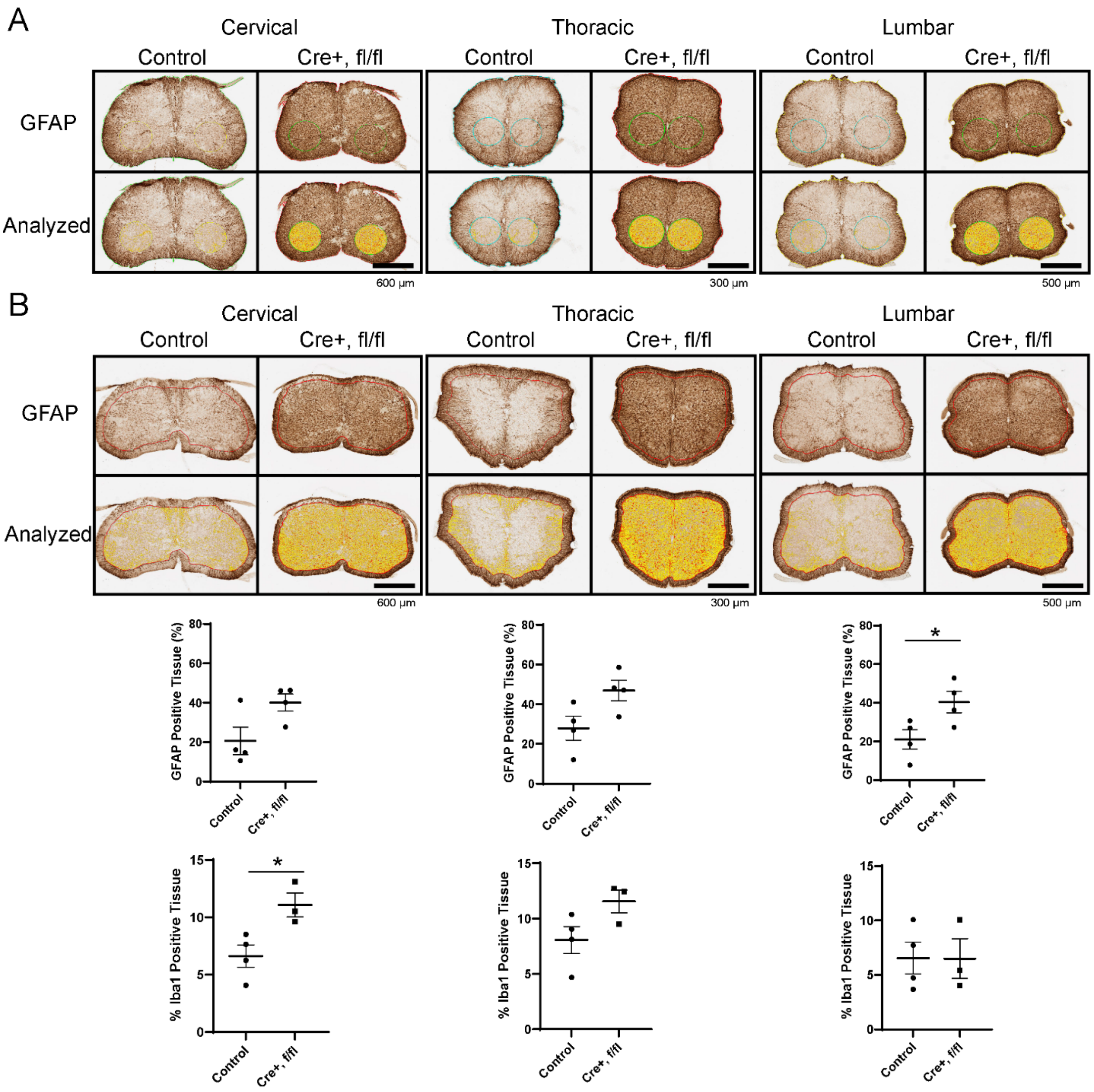

Figure S4. Methodology for quantification of GFAP- and Iba1-positive immunoreactivity in spinal cord sections of VPS35 cKO mice at P12-14 using HALO analysis software. (**A**) Analysis of total area occupied by GFAP staining in the ventral horns of cervical, thoracic or lumbar spinal cord (highlighted by HALO in the green circle area of analyzed sections, using yellow, orange and red to indicate the intensity levels of staining). This method was employed to quantify data shown in Figure 9. (**B**) Analysis of total area occupied by GFAP or Iba1 staining in gray matter regions of whole cervical, thoracic or lumbar spinal cord sections. A border (in red) was defined for each section to exclude quantification of dense non-specific staining at the section periphery, as indicated. Graphs indicate the quantitation of total tissue area occupied by GFAP

(top row) or Iba1 (bottom row) immunoreactivity in whole spinal cord sections of *VPS35$^{fl/fl}$/Cre* and control mice. Bars represent mean ± SEM (*n* = 4 mice/genotype for GFAP, or *n* = 3-4 mice/genotype for Iba1). *P<0.05 by unpaired, two-tailed Student's *t*-test.

Supplementary Videos:

Supplementary Video 1. Motor phenotypes of *VPS35 cKO/Syn1-Cre* mice at P10. Video showing two *VPS35$^{fl/fl}$/Cre* mice (located left and top of box at the beginning). Control littermate mice are clustered below (3x *VPS35$^{fl/fl}$,* 1x *VPS35$^{fl/wt}$,* 1x *VPS35$^{fl/wt}$/Cre*).

Supplementary Video 2. Motor phenotypes of *VPS35 cKO/Syn1-Cre* mice at P15. Video closely highlighting the motor symptoms of a *VPS35$^{fl/fl}$/Cre* mouse at P15.

Supplementary Tables:

Table S1. Pairwise comparisons of *VPS35* expression between different mouse brain cell types by single-cell RNAseq with Kruskal-Wallis multiple comparison.

Dunn's multiple comparisons test	Mean rank diff.	Significant?	Summary	Adjusted P Value
Neurons vs. Enteric Glia	71.39	No	ns	0.5787
Neurons vs. OPC/Oligo	14.69	No	ns	>0.9999
Neurons vs. Neuroblast/neurogliaform	88.66	Yes	***	0.0001
Neurons vs. ependyma	54.77	No	ns	>0.9999
Neurons vs. vascular	117.1	Yes	**	0.002
Neurons vs. pericyte	94.72	No	ns	>0.9999
Neurons vs. microglia	128.7	No	ns	0.136
Neurons vs. Satellite glia	80.22	No	ns	>0.9999
Neurons vs. astrocytes	128.4	Yes	**	0.0015
Enteric Glia vs. OPC/Oligo	-56.7	No	ns	>0.9999
Enteric Glia vs. Neuroblast/neurogliaform	17.26	No	ns	>0.9999
Enteric Glia vs. ependyma	-16.63	No	ns	>0.9999
Enteric Glia vs. vascular	45.71	No	ns	>0.9999
Enteric Glia vs. pericyte	23.33	No	ns	>0.9999
Enteric Glia vs. microglia	57.33	No	ns	>0.9999
Enteric Glia vs. Satellite glia	8.833	No	ns	>0.9999
Enteric Glia vs. astrocytes	57	No	ns	>0.9999
OPC/Oligo vs. Neuroblast/neurogliaform	73.96	No	ns	0.5795
OPC/Oligo vs. ependyma	40.08	No	ns	>0.9999
OPC/Oligo vs. vascular	102.4	No	ns	0.2409
OPC/Oligo vs. pericyte	80.03	No	ns	>0.9999
OPC/Oligo vs. microglia	114	No	ns	0.9119
OPC/Oligo vs. Satellite glia	65.53	No	ns	>0.9999
OPC/Oligo vs. astrocytes	113.7	No	ns	0.1427
Neuroblast/neurogliaform vs. ependyma	-33.89	No	ns	>0.9999
Neuroblast/neurogliaform vs. vascular	28.45	No	ns	>0.9999
Neuroblast/neurogliaform vs. pericyte	6.069	No	ns	>0.9999
Neuroblast/neurogliaform vs. microglia	40.07	No	ns	>0.9999
Neuroblast/neurogliaform vs. Satellite glia	-8.431	No	ns	>0.9999
Neuroblast/neurogliaform vs. astrocytes	39.74	No	ns	>0.9999
ependyma vs. vascular	62.34	No	ns	>0.9999
ependyma vs. pericyte	39.96	No	ns	>0.9999
ependyma vs. microglia	73.96	No	ns	>0.9999
ependyma vs. Satellite glia	25.46	No	ns	>0.9999
ependyma vs. astrocytes	73.63	No	ns	>0.9999
vascular vs. pericyte	-22.38	No	ns	>0.9999
vascular vs. microglia	11.62	No	ns	>0.9999
vascular vs. Satellite glia	-36.88	No	ns	>0.9999
vascular vs. astrocytes	11.29	No	ns	>0.9999
pericyte vs. microglia	34	No	ns	>0.9999
pericyte vs. Satellite glia	-14.5	No	ns	>0.9999
pericyte vs. astrocytes	33.67	No	ns	>0.9999
microglia vs. Satellite glia	-48.5	No	ns	>0.9999
microglia vs. astrocytes	-0.3333	No	ns	>0.9999
Satellite glia vs. astrocytes	48.17	No	ns	>0.9999

Table S2. Pairwise comparisons of *VPS35* expression between mouse brain neuronal populations by single-cell RNAseq with Kruskal-Wallis multiple comparison.

Dunn's multiple comparisons test	Mean rank diff.	Significant?	Summary	Adjusted P Value
Neuroblast/radial glial vs. Non-neuron	-8.304	No	ns	>0.9999
Neuroblast/radial glial vs. Glutamatergic	-86.82	Yes	**	0.0034
Neuroblast/radial glial vs. GABAergic	-42.75	No	ns	>0.9999
Neuroblast/radial glial vs. Cholinergic	-149.3	Yes	****	<0.0001
Neuroblast/radial glial vs. Noradrenergic	-150.7	Yes	***	0.0003
Neuroblast/radial glial vs. Peptidergic DRG	-132.6	Yes	**	0.0033
Neuroblast/radial glial vs. Non-peptidergic DRG	-168.1	Yes	****	<0.0001
Neuroblast/radial glial vs. Serotonergic	-176.7	Yes	***	0.0003
Neuroblast/radial glial vs. MSNs	-9.845	No	ns	>0.9999
Non-neuron vs. Glutamatergic	-78.52	Yes	****	<0.0001
Non-neuron vs. GABAergic	-34.45	No	ns	0.6575
Non-neuron vs. Cholinergic	-141	Yes	****	<0.0001
Non-neuron vs. Noradrenergic	-142.4	Yes	****	<0.0001
Non-neuron vs. Peptidergic DRG	-124.3	Yes	***	0.0007
Non-neuron vs. Non-peptidergic DRG	-159.8	Yes	****	<0.0001
Non-neuron vs. Serotonergic	-168.4	Yes	****	<0.0001
Non-neuron vs. MSNs	-1.542	No	ns	>0.9999
Glutamatergic vs. GABAergic	44.07	Yes	*	0.0182
Glutamatergic vs. Cholinergic	-62.45	No	ns	0.1185
Glutamatergic vs. Noradrenergic	-63.86	No	ns	>0.9999
Glutamatergic vs. Peptidergic DRG	-45.73	No	ns	>0.9999
Glutamatergic vs. Non-peptidergic DRG	-81.3	No	ns	0.1012
Glutamatergic vs. Serotonergic	-89.86	No	ns	0.4469
Glutamatergic vs. MSNs	76.98	No	ns	0.7283
GABAergic vs. Cholinergic	-106.5	Yes	****	<0.0001
GABAergic vs. Noradrenergic	-107.9	Yes	**	0.0057
GABAergic vs. Peptidergic DRG	-89.8	No	ns	0.064
GABAergic vs. Non-peptidergic DRG	-125.4	Yes	***	0.0001
GABAergic vs. Serotonergic	-133.9	Yes	**	0.0057
GABAergic vs. MSNs	32.9	No	ns	>0.9999
Cholinergic vs. Noradrenergic	-1.406	No	ns	>0.9999
Cholinergic vs. Peptidergic DRG	16.72	No	ns	>0.9999
Cholinergic vs. Non-peptidergic DRG	-18.85	No	ns	>0.9999
Cholinergic vs. Serotonergic	-27.41	No	ns	>0.9999
Cholinergic vs. MSNs	139.4	Yes	**	0.0051
Noradrenergic vs. Peptidergic DRG	18.13	No	ns	>0.9999
Noradrenergic vs. Non-peptidergic DRG	-17.44	No	ns	>0.9999
Noradrenergic vs. Serotonergic	-26	No	ns	>0.9999
Noradrenergic vs. MSNs	140.8	Yes	*	0.0248
Peptidergic DRG vs. Non-peptidergic DRG	-35.57	No	ns	>0.9999
Peptidergic DRG vs. Serotonergic	-44.13	No	ns	>0.9999
Peptidergic DRG vs. MSNs	122.7	No	ns	0.1176
Non-peptidergic DRG vs. Serotonergic	-8.556	No	ns	>0.9999
Non-peptidergic DRG vs. MSNs	158.3	Yes	**	0.0031
Serotonergic vs. MSNs	166.8	Yes	*	0.0118

Table S3. Pairwise comparisons of *VPS35* expression between neurons of different anatomic mouse brain regions by single-cell RNAseq with Kruskal-Wallis multiple comparison.

Dunn's multiple comparisons test	Mean rank diff.	Significant?	Summary	Adjusted P Value
Non-neurons vs. CB	50.38	No	ns	>0.9999
Non-neurons vs. FC	-59.44	No	ns	0.8345
Non-neurons vs. PC	-11.45	No	ns	>0.9999
Non-neurons vs. ENT	104.4	No	ns	>0.9999
Non-neurons vs. GP	140.5	Yes	****	<0.0001
Non-neurons vs. HC	-90.89	Yes	**	0.0012
Non-neurons vs. STR	-178.3	Yes	****	<0.0001
Non-neurons vs. SN	41.34	No	ns	>0.9999
Non-neurons vs. TH	101.7	No	ns	0.0824
CB vs. FC	-109.8	No	ns	>0.9999
CB vs. PC	-61.83	No	ns	>0.9999
CB vs. ENT	54.07	No	ns	>0.9999
CB vs. GP	90.13	No	ns	>0.9999
CB vs. HC	-141.3	No	ns	0.5183
CB vs. STR	-228.6	Yes	*	0.0112
CB vs. SN	-9.039	No	ns	>0.9999
CB vs. TH	51.32	No	ns	>0.9999
FC vs. PC	47.99	No	ns	>0.9999
FC vs. ENT	163.9	No	ns	0.0593
FC vs. GP	199.9	Yes	****	<0.0001
FC vs. HC	-31.45	No	ns	>0.9999
FC vs. STR	-118.8	No	ns	0.1598
FC vs. SN	100.8	No	ns	0.2819
FC vs. TH	161.1	Yes	**	0.0013
PC vs. ENT	115.9	No	ns	0.8857
PC vs. GP	152	Yes	***	0.0001
PC vs. HC	-79.44	No	ns	0.1735
PC vs. STR	-166.8	Yes	***	0.0009
PC vs. SN	52.79	No	ns	>0.9999
PC vs. TH	113.1	No	ns	0.0942
ENT vs. GP	36.06	No	ns	>0.9999
ENT vs. HC	-195.3	Yes	**	0.0034
ENT vs. STR	-282.7	Yes	****	<0.0001
ENT vs. SN	-63.11	No	ns	>0.9999
ENT vs. TH	-2.753	No	ns	>0.9999
GP vs. HC	-231.4	Yes	****	<0.0001
GP vs. STR	-318.8	Yes	****	<0.0001
GP vs. SN	-99.17	No	ns	0.473
GP vs. TH	-38.81	No	ns	>0.9999
HC vs. STR	-87.37	No	ns	>0.9999
HC vs. SN	132.2	Yes	**	0.0057
HC vs. TH	192.6	Yes	****	<0.0001
STR vs. SN	219.6	Yes	****	<0.0001
STR vs. TH	279.9	Yes	****	<0.0001
SN vs. TH	60.36	No	ns	>0.9999

Uncropped Southern blot

From Figure 1E

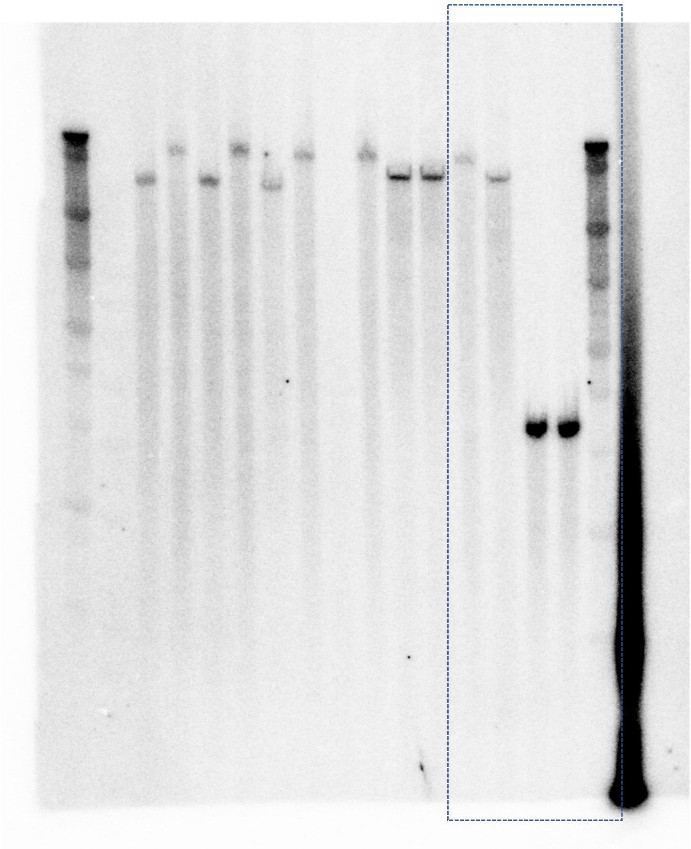

Uncropped genotyping PCR agarose gel

From Figure 1F

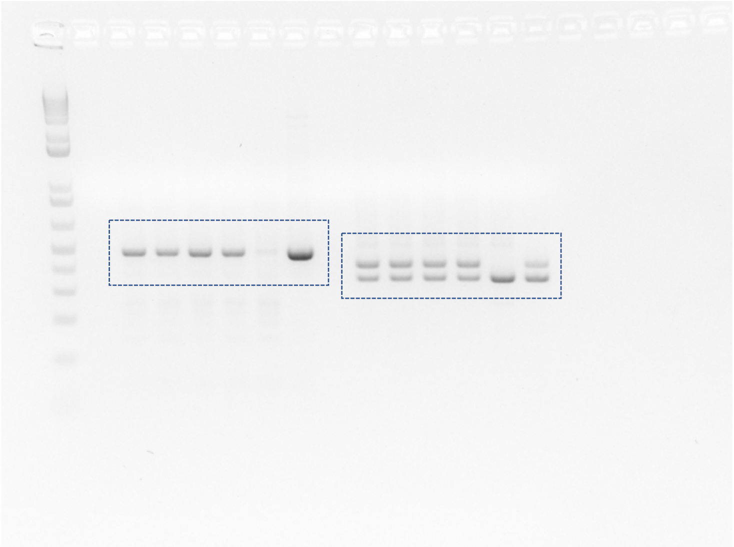

Uncropped Western blots.

From Figure 1G.

VPS35

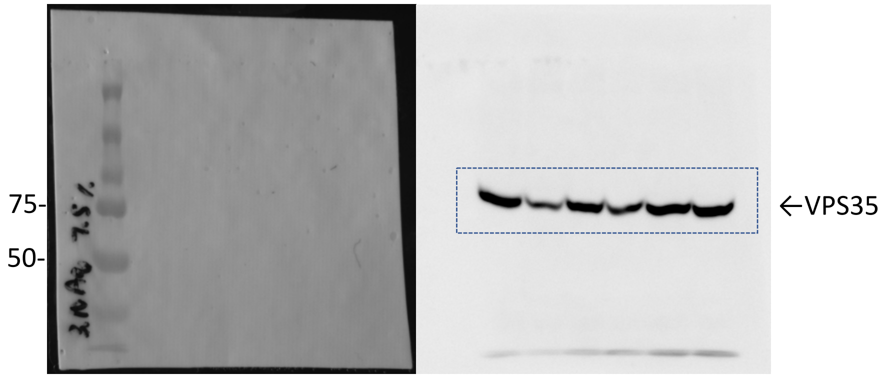

VPS26

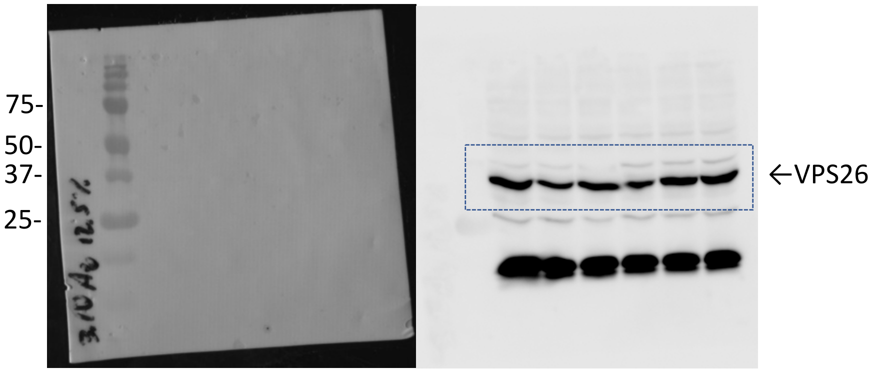

VPS29

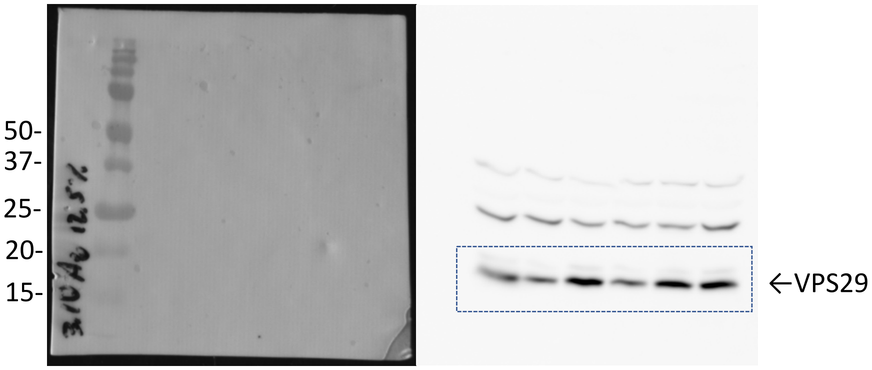

Actin

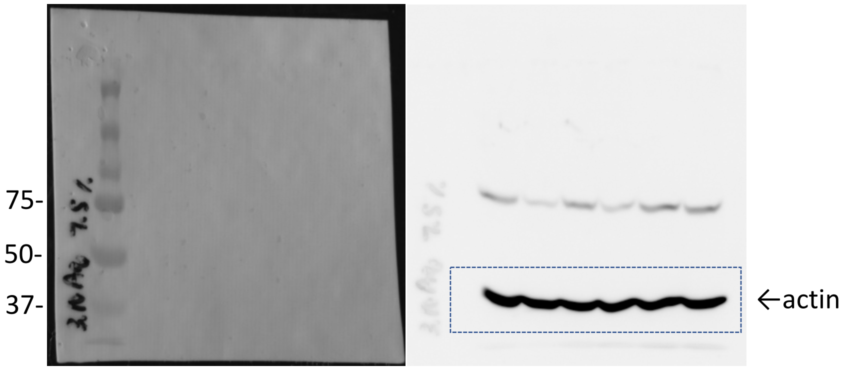

Uncropped Western blots.

From Figure 2F.

VPS35

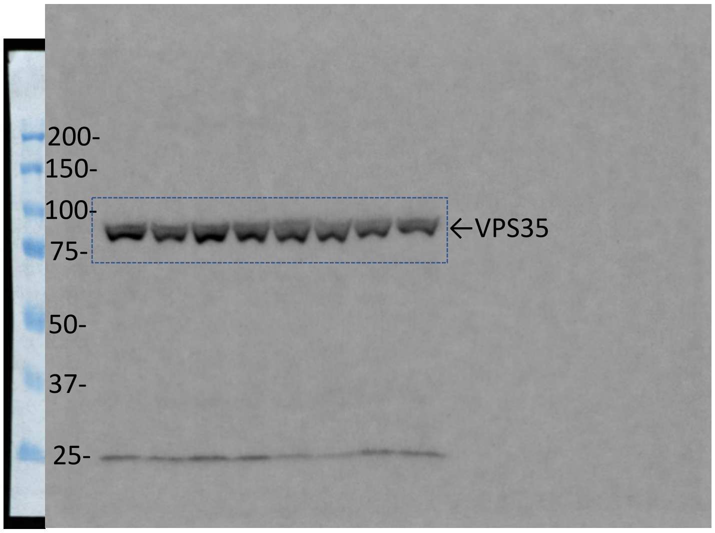

VPS26

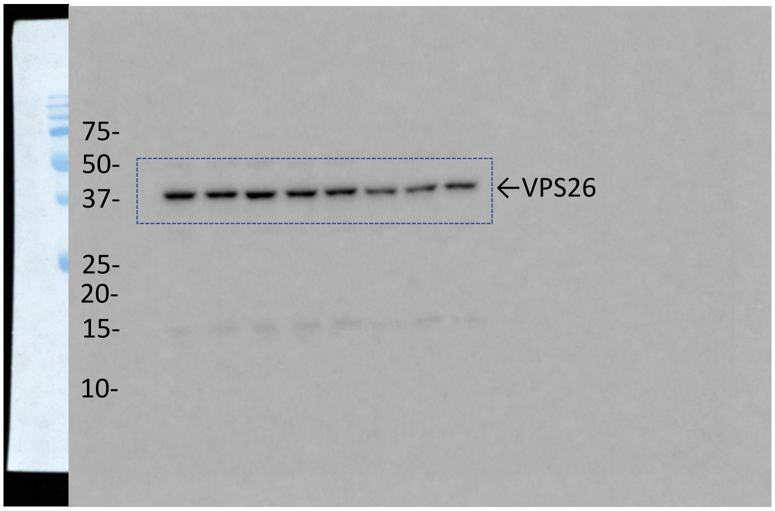

VPS29

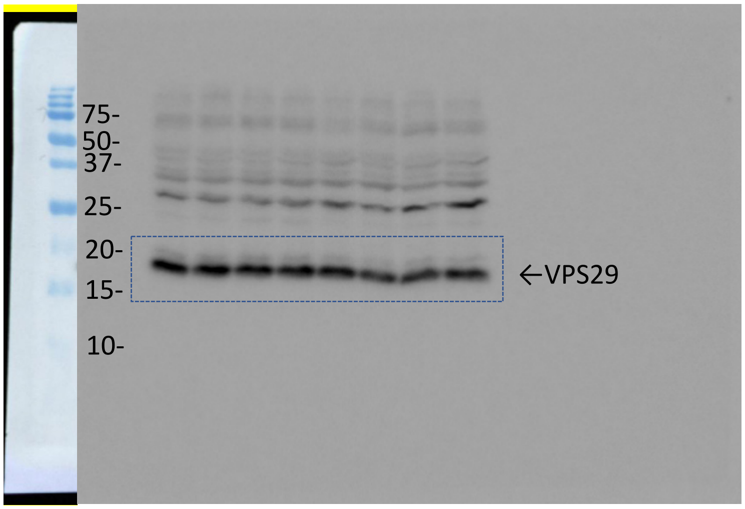

Actin

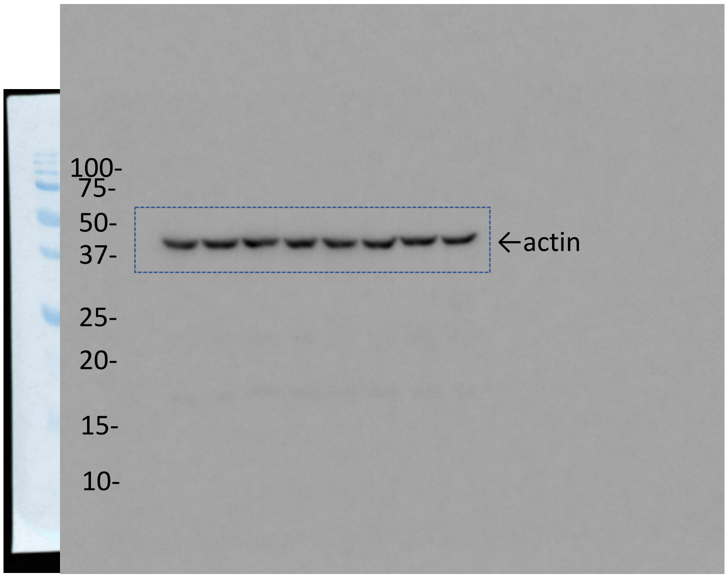

Supplement: fcab208_Supplementary_Data [file fcab208_supplementary_data.zip › Supplementary Material.pdf]
